# Topological rules and anomalies in intramolecular G-quadruplex folding: a comprehensive study

**DOI:** 10.1093/nar/gkag435

**Published:** 2026-05-19

**Authors:** Anton Granzhan, Liliane Mouawad

**Affiliations:** Laboratoire de Chimie et Biochimie Pharmacologiques et Toxicologiques (LCBPT), CNRS UMR8601, Université Paris Cité, Paris 75006, France; Chemistry and Modeling for the Biology of Cancer, CNRS UMR9187, INSERM U1196, Institut Curie, PSL Research University, Université Paris-Saclay, Orsay 91400, France

## Abstract

G-quadruplexes (G4s) make knots in DNA and RNA. One-block G4s can adopt eight different topologies, yet their folding rules are not precisely known. A systematic study of 353 intramolecular G4s enabled us to establish certain characteristics that rule their topology. In the absence of restraints imposed by a ligand or modified guanosines, when two loops are one nucleotide-long (nt), the topology is parallel, regardless of the third loop length. When loops 1 and 3 are 2-nt long, loop 2 length (L_2_) determines the G4 topology: parallel for L_2_ = 1, parallel or antiparallel-chair for L_2_ = 2, antiparallel-chair for L_2_ = 3, and antiparallel-basket for L_2_ = 4–5 nts. Diagonal loops require at least 4 nts; the only three structures with a diagonal loop of 3 nts present multiple anomalies. Only three topologies can accommodate a diagonal loop; the other five cannot. This shows the importance of distinguishing between the eight different topologies. We explain why parallel and hybrid topologies form preferentially three-tetrad G4s, while antiparallel topologies form preferentially two-tetrad G4s. We also explain why bulges between the G4 stem and the hairpin loop in quadruplex-duplex hybrids are sometimes needed. These findings clarify G4 folding principles and topological restraints.

## Graphical Abstract

**Graphical Abstract:**
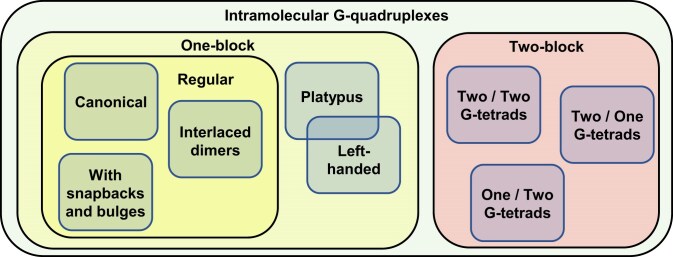

## Introduction

G-quadruplex (G4) is a non-canonical structure found in G-rich DNA and RNA sequences. Numerous G4-forming sequences are found in telomeres [1–3], oncogene promoters [4–6], intronic regions [7, 8], etc. Therefore, they constitute a potential drug target for aging and several diseases, including cancer, infectious, and neurological diseases [5, 9, 10].

**Figure 1.**
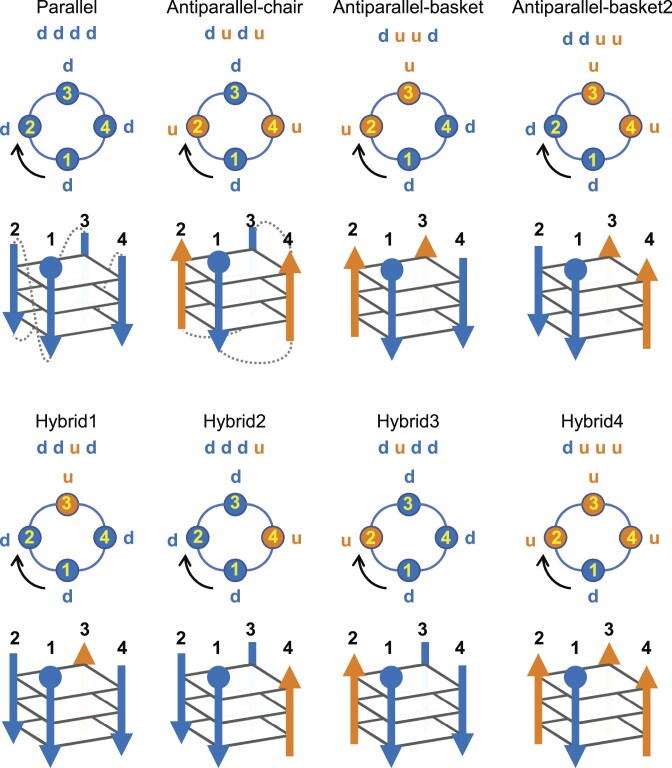
Schematic representation of the eight distinct topologies as given by ASC-G4, top and side views. The structure is considered from top to bottom. The strands are all numbered clockwise according to the direction of the black curved arrow. The strand directions down (d) are colored blue and up (u), orange. In the side view, the beginning of the stem is represented by a filled blue circle; in the first two topologies, the loops are drawn as dashed lines. They are omitted in the rest for clarity. In the antiparallel-chair topology, the strand numbering does not follow the chain; here, the first loop connects strands 1 and 4, which are the two adjacent G-tracts in the chain.

G4s consist of two to four guanine tetrads (G-tetrads), each containing four guanines, related by Hoogsteen interactions. The intramolecular G4 is formed by a single nucleotide chain, which can fold in one or two blocks. It can adopt eight different topologies, which are grouped in either parallel, antiparallel, or hybrid, depending on the mutual direction of the strands. The strands are the edges of the G-tetrads. When a G4 is viewed from top to bottom (generally, from 5′-tetrad to 3′-tetrad), the first strand direction is down (d), and the others are either down (d) or up (u). In this orientation, when the strands are numbered clockwise, the eight topologies, which are expected theoretically, can be unambiguously distinguished. They are parallel with the four strands down (dddd), antiparallel-chair (dudu), antiparallel-basket (duud), antiparallel-basket2 (dduu), with two strands down and two strands up, and hybrid1 (ddud), hybrid2 (dddu), hybrid3 (dudd), and hybrid4 (duuu), with three strands in opposite directions to the fourth (Fig. 1). In the two-block structures, there are discontinuities in at least three of the four strands, between the same two tetrads, delimiting the blocks. Each block can have its own topology among the eight mentioned above, but all structures that were resolved to date adopt the parallel/parallel topology, parallel/hybrid2, −/parallel, and parallel/−, where ‘−‘ stands for the absence of topology because the block consists of a single G-tetrad; the block that has a topology always consists of two tetrads. In all G4s, there may be discontinuities in the strands due to bulges, snapbacks, or internal loops (D-loops) (Supplementary Fig. S1 in the Supplementary Data). The latter mainly link the two separate blocks in the two-block structures, whereas bulges and snapbacks concern both the one-block and two-block G4s. A bulge consists of one or several nucleotides (nts), up to 15 to date (7CLS [11]), that insert within a strand’s sequence, without making part of the G4 stem (or core). A snapback consists of one or two guanines that snap back after (or before) a relatively long snapback loop (between 1 and 17 nts to date) to insert in a different strand within the stem. Four types of snapbacks were identified: 5′-bottom, 5′-top, 3′-bottom, and 3′-top, where 5′ and 3′ refer to the extremity of the stem chain that snaps back (excluding the overhangs if they exist) and bottom and top refer to the tetrad, which includes the snapback [12, 13].

As presented above, intramolecular G4s can be separated into two sets of structures: the one-block set and the two-block set. Irrespectively of their topology, G4s can be classified into different subsets. The simplest is the two-block set, which consists of three disjoint subsets: 1) the structures made of two/two G-tetrads, 2) two/one G-tetrads, and 3) one/two G-tetrads (refer to the Graphical Abstract). The one-block set is far more complex; it consists of regular G4s, platypus, and left-handed G4s. The platypus G4s combine features from several topologies due to particularities in their sequences [13]; they can be either right-handed (RH) or left-handed (LH) structures. The LH subset can contain platypus or normal G4 structures; thus, the LH and platypus subsets are not disjoint. The set of regular G4s consists of the one-block RH structures, which have their characteristics belonging to a well-defined topology. This set consists of three disjoint subsets: 1) canonical structures, 2) those with discontinuities, and 3) interlaced dimers. The canonical G4s have no discontinuities in the strands, like bulges or snapbacks. They can be either monomers or stacking-stem dimers, meaning that the stems of the two monomers stack on each other. The second subset consists of structures with discontinuities, which can also be monomers or dimers, whereas the third subset is made only of interlaced dimers. In the latter, the top tetrad of each monomer contains three guanosines from this monomer, while the fourth one belongs to the other monomer.

The strands are connected by three types of loops. The usual definition of the loops is the following: 1) *propeller*, a loop that connects two parallel adjacent strands; 2) *lateral*, a loop that connects two antiparallel adjacent strands; 3) *diagonal*, a loop that connects two antiparallel opposite strands. In addition, there are two other scarce types of loops: the D-shaped loop, which we also call the internal loop, connects two nucleotides in the same strand going from bottom to top; apart from one exception (2M53 [14]), it is only found in the two-block structures. In ASC-G4 [12], which was used in this study, this loop is called ‘linker’ because it links the two blocks (Supplementary Fig. S1). The V-shaped loop is more problematic; it is generally defined to consist of 0 nts [15, 16], but was also described to include more nts [17, 18]. We will show below that the V shape is not a feature of the loop, but of the strand that follows the 0-nt loop. This is the reason why we adopt the more general definition of the loops, which is independent of their length or the direction of the strands: 1) the *propeller* loop, connecting two nts from adjacent strands and different tetrads; 2) the *lateral* loop, connecting two nts from adjacent strands and the same tetrad; 3) the *diagonal* loop, connecting two nts from two opposite strands and the same tetrad, 4) the *internal loop* (*linker*, or *D-shaped* loop), mostly connecting the two blocks in the two-block G4s. This definition of propeller, lateral, and diagonal loops, as well as the linker, is adopted in ASC-G4 (http://tiny.cc/ASC-G4 or https://institut-curie.vksolutions.io/asc-g4), which was used here to analyze the structures of intramolecular G4s.

Some G4-forming sequences are known to be polymorphic. Their fold can depend on pH [19], the type of coordinated cations [20–22], solution crowding [23–25], flanking nucleotides (FN) or overhangs [20], temperature [26], and ligands [27–29]. We may ask if all G4 sequences are polymorphic, and what the role of the loops and FNs is in the adopted topology. Numerous studies attempted to answer these questions, using various biophysical or computational methods [30–35]. G4ShapePredictor is an AI-based software trained to predict G4 topology (just distinguishing between parallel, antiparallel, or hybrid) for sequences optimally containing 20–24 nts [36]. Although it has been described to produce rather good results, it does not always succeed, even for canonical structures, probably because G4 folds are too complex despite the small size of the system. A knowledge of the loops may be exploited to predict possible G4 topologies, or exclude others, but the rules for this have not yet been established. Herein, we provide some clues regarding the role of the loops and overhangs on the G4 topology, based on the comprehensive analysis of 353 intramolecular structures available in the Protein Data Bank (PDB) (http://www.rscb.org) [37]. The results of our study are not intended to allow the prediction of G4 topologies, but rather to establish a set of rules governing the possibility of a given sequence to adopt certain topologies, or to exclude certain others. Following these rules, some sequences are shown to have a robust topology, unaffected by exogenous conditions.

## Materials and methods

### ASC-G4 analysis

ASC-G4 was applied to the 353 intramolecular G4 structures [12] (http://tiny.cc/ASC-G4 or https://institut-curie.vksolutions.io/asc-g4). The results include all advanced G4 characteristics, among which are the sequence, topology, loops, presence of bulges, snapbacks, and linkers (i.e. the D-loops in the two-block structures), stem guanine identifications and glycosidic configurations (gc), rise, tilt, and twist angles, groove widths, minimum groove widths, and so on. From all this, the needed information was extracted using home-made programs. Selected ASC-G4 results for all examined sequences, i.e. the main characteristics needed in this study, are gathered in an Excel file provided as Supplementary Data. The results are grouped by topologies in Supplementary Table S1 for the one-block G4s, and Supplementary Table S2, for the two-block G4s. In Supplementary Tables S1 and S2, the PDB IDs of all structures are provided, but without their references, considering their large number. However, these references can be easily accessed via the ASC-G4 website, by choosing “Intramolecular G4” → “Upload PDB structure” → “Search PDB file in RCSB PDB” → Enter PDB ID → “Access article”.

All structural images were drawn using the Visual Molecular Dynamics (VMD) software [38], and the plots, using Gnuplot (http://www.sourceforge.net/projects/gnuplot).

## Results and discussion

### Generalities regarding the G4 structures

We analyzed 353 intramolecular G4 structures available from the PDB. They correspond to 218 non-redundant sequences: 192 forming one-block G4s and 26 two-block G4s. Here, the sequences are considered redundant when they are identical; the modification of one nt suffices to remove the redundancy. However, unless otherwise stated, all redundant sequences are considered because the corresponding structures may depend on the experimental conditions (ligands, ions, pH…). ASC-G4 [12] was applied to all 353 structures to calculate their advanced characteristics. Generally, when a G4 structure is resolved, several related sequences are studied in parallel, using biophysical methods. These sequences were not included herein because their topology is not known with certainty, except when the 3D structure is resolved, and even then, doubts remain in some cases, as will be shown below. Usually, CD spectroscopy is used to determine the topology of a G4 with an unknown structure. Yet, CD spectra only reflect the presence of homopolar stacking (peak at 260 nm) and heteropolar stacking of guanines (peak at 297 nm). However, heteropolar stacking can exist in parallel structures, like those with an all-*syn* first tetrad (6ERL [34]), and homopolar stacking can exist in an antiparallel structure, when, for instance, there is an additional G-triad (2KF8 [39]). This makes these “hybrid” CD spectra misleading. Besides, significantly decreasing the stability of a sequence due to guanine modifications does not necessarily imply a change of topology. For instance, introducing *syn-*Gs in a parallel Myc sequence destabilizes the structure by decreasing its melting temperature by more than 40°C, without altering the topology (according to the CD spectra) or the number of tetrads (according to the 1D ^1^H NMR spectra) [34]. Because of these uncertainties, we only focus on the resolved structures deposited in the PDB.

### Characteristics of the one-block G4 structures

The results grouped by topologies are summarized in Table 1 and Supplementary Table S1 for the one-block G4s. In Table 1, the total number of non-redundant one-block G4 sequences is 197 instead of the 192 mentioned above. This is because, in the table, the sequences are categorized according to the topology of the 3D structures, while there are three sequences corresponding to two different topologies and one sequence corresponding to 3 different topologies. Three of these four sequences are human telomeric G4s (htel): 1) *22AG*: d[AG_3_(TTAG_3_)_3_] is antiparallel-basket (143D [40]) and parallel (1KF1 [1]), depending on the presence of a crowding agent and the metal cation (Na^+^ versus K^+^); 2) *TAGGG*: d[TAG_3_(TTAG_3_)_2_TTAG_2_] can be antiparallel-basket (8JIC [41]) or antiparallel-basket2 (8JIH [41]) depending on pH; 3) *23TAG*: d[TAG_3_(TTAG_3_)_3_] can be hybrid1 (2JSM [42]), when free and antiparallel-chair (7Z9L [28]), in complex with Phen-DC3, or parallel (2LD8 [43]), in the presence of a crowding agent. The fourth sequence is from the *RET* promoter, *RET20T*: d[(G_4_C)_3_G_4_T], which is parallel, with an all-*syn* first tetrad (2L88 [44]) in K^+^, or hybrid4 (7YS5 [45]) in Na^+^. Recently, two published structures, which are not in our set, show that one or two Pt−phen ligands transform the parallel structure of *MYC1234*: d[TA(G_3_AG_3_TAG_3_AG_3_T] (2LBY [46] and 5LIG [47]) into hybrid1 (9B6Z and 8YU3 [48]). Curiously, considering the well-known polymorphism of G4s, we could expect that more sequences would lead to multiple topologies among the resolved structures due to extrinsic conditions (which are different from mutations or guanine modifications). Contrary to the polymorphic sequences listed above, others can be very robust and do not change their topology, despite some drastic modifications. They are mainly parallel or antiparallel-chair. This is the case of the *MYC22* mutant, d[TGA(G_3_T)_2_A(G_3_T)_2_AA], which is always parallel (1XAV [49]), whether the last T is replaced with G (7KBV [50]), the first G of each tract is replaced with ^Br^G, promoting a *syn* configuration (6ERL), a long 5′-FN (6ZL9 [51]), a long 3′-FN (6ZL2 [51]) or both (8DUT [52]) are added. This is remarkable considering that the structures of *MYC22* were resolved using either NMR, X-ray crystallography, or electron microscopy (EM). Similarly, the sequences d[(GGA)_4_] and r[(GGA)_4_] are parallel dimers whether they be DNA (1MYQ [53]) or RNA (2RQJ [54]), and d[(G_2_T_2_G_2_)TGT(G_2_T_2_G_2_)] is always anti-parallel-chair (148D [55]), in all resolved structures, whatever the cation (1BUB [56]), the crowding agent (7ZKM [57]), the mutations (7D33 [58]) or the length of the FNs (5CMX [59]). Only robust topologies will be discussed below, whereas the polymorphism of telomeric G4 will be treated in an upcoming article.

**Table 1.** Characteristics of the 318 one-block structures. In the first column, for each topology, are given the number of structures (in bold) and the number of non-redundant sequences (in roman). In the following columns are given the direction of the strands (d for down and u for up), the main glycosidic configuration (gc) patterns (a for *anti*-G and s for *syn*-G), the nature of the nucleotide chains (DNA or RNA), and the groove width signatures (w for wide, n for narrow, and i for intermediate or medium groove). In columns 6–8 are reported, for each topology, the number of structures with 2, 3, or 4 tetrads, respectively, in each monomer. In the last column, for each topology, the loop combinations are ordered from the highest occurrence to the lowest (p for propeller, l for lateral, and d for diagonal, + and − are the loop progression, clockwise or anticlockwise, respectively). The occurrence (in parentheses) is the number of structures with the corresponding characteristic. In the presence of a snapback, the quadruplex has four loops. Of note, the loop order in each combination follows the nucleotide chain sequence, and its progression is determined by looking at the G4 stem from bottom to top, whereas the order of the strand directions, the gcs, and the groove widths follows the Hoogsteen pairing in the clockwise direction, determined by looking at the G4 stem from top to bottom

Topology(number of structures) (number of non-redundant sequences)	Strand directions	Main gc patterns	Nature of the chain(number of structures) (number of non-redundant sequences)	Groove-width signatures	Number of structures with			Loop combinations (occurrence)
					2 tetrads	3 tetrads	4 tetrads	
**Parallel**(**140**) (91)	dddd	aaaa ssss	DNA (**125**) (81)	iiii	4	116	5	−p−p−p(104), −p−p−p*d*^a^(7),−p−p−l*+p*(5), −p−p−p−*l*(4),*−p*−p−p−p(1), *−p*−p−p−*p*(1),−p−p−p+*l*(1), −p*i*^b^*−*l−p(1),+*l*−p−p−p(1)
			RNA (**15**) (10)		9	6	0	−p−p−p(15)
**Antiparallel-chair**(**64**) (22)	dudu	sasa asas	DNA (**64**) (22)	wnwn	57	5	2	+l+l+l(54), −l−l−l(9),*+l*−l−p−l(1)
**Antiparallel-basket**(**24**) (21)	duud	saas assa	DNA (**24**) (21)	wini	12	8	4	−ld+l(22), d+pd(2)
**Antiparallel-basket2**(**16**) (10)	dduu	ssaa aass	DNA (**7**) (5)	iwin	3	3	1	+ld−l(6), +l+p+l(1)
			RNA (**9**) (5)	iwii	9	0	0	−pd+p(9)
**Hybrid1**(**16**) (12)	ddud	ssas aasa	DNA (**16**) (12)	iwni	1	14	1	−p−l−l(14), *+l*−p−l−l(1),−p−l−p(1)
**Hybrid2**(**17**) (14)	dddu	sssa aaas	DNA (**10**) (9)	iiwn	1	9	0	−pd+l(7), −p−p−l(1),+ld−p(1), −l−p−l*d* (1)
			RNA (**6**) (4)		6	0	0	−p−p−l(6)
			DNA–RNA (1)		0	1	0	−pd+l(1)
**Hybrid3**(**24**) (15)	dudd	sass asaa	DNA (**20**) (14)	wnii	1	19	0	−l−l−p(20)
		aaaa	RNA (**4**) (1)		4	0	0	*−l*+l+p+l(4)
**Hybrid4**(**17**) (13)	duuu	saaa asss	DNA (**17**) (13)	wiin	0	17	0	*−l*+l+p+p(8), *d*+p+l+p(4),+l+p+p(3), +l+p+p+*l*(2)

^a^Snapback loops are in italics: at the beginning of the combination, they correspond to 5′-snapbacks (5′-bottom or 5′-top), and at the end, to 3′-snapbacks (3′-bottom or 3′-top)

^b^The exceptional internal (*i*) or D-loop, which goes from bottom to top in strand 2, found in one structure, 2M53 [14]

In Table 1, the one-block structures are mostly made of three tetrads when their topology is either parallel or hybrid, but mostly of two tetrads when the topology is antiparallel. A few 2-tetrad structures are found in parallel, hybrid2, and hybrid3 topologies, but none in hybrid4, and only one in hybrid1. The latter is a particular structure, 1JJP [17], which is a 2-tetrad DNA interlaced dimer, and its topology could not be determined from the direction of its strands because of the discontinuity in one strand, so it was determined from its gc pattern and groove-width signature [13]. All the other known interlaced dimers consist of three tetrads in each monomer, and their topology is parallel. Three G4-DNA structures have one 3-G top tetrad, completed with a G-like extrinsic molecule (6K3Y [60], 6V0L [61], and 7WGW [62]). This tetrad was not detected by ASC-G4, and therefore, these structures were given as a 2-tetrad parallel. However, in Table 1, they are counted as 3-tetrad G4s. Most of the G4-DNA hybrid2 structures consist of three tetrads and contain a diagonal loop, except 6JCD [63], which is made of two tetrads, with the loop progression −l−p−l*d*, and 7O1H [64], which consists of three tetrads but does not have any diagonal loop (loop progression −p−p−l), because of the shortness of its first two loops (1 nt), only possible as propeller loops (see subsection “*The loops and their influence on topology*” below).

All the G4-RNA hybrid2 and hybrid3 structures are made of two tetrads, whereas only one G4-DNA is a 2-tetrad hybrid2 (6JCD [63], with a 3′-top snapback) and one other G4-DNA, a 2-tetrad hybrid3 (2MFU, unpublished). Whatever the topology, most of the one-block G4-RNAs (28 of 34 G4-RNAs) contain two tetrads; the six remaining G4-RNAs consist of 3-tetrad parallel structures (their chains are 22–23 nts long). This may seem contradictory with the main assertion reported in [65] that 2-tetrad G4-RNAs are unstable unless they dimerize, but it is not. First, in the cited article, the authors acknowledge the presence of 2-tetrad G4-RNA within long sequences (typically aptamers). Second, the RNA sequences studied in [65] are short (not exceeding 21 nts), and the assertion about the two tetrads only holds for this chain length, even though it is not explicitly specified. In our set of resolved 2-tetrad G4-RNA structures, the short chains (up to 14 nts, 4 structures) also dimerize; however, when the chain is long enough (between 36 and 374 nts, 24 structures), the overhangs and the loops form either one or two RNA duplexes above and below the G4 core, thereby stabilizing the 2 tetrads. Therefore, in nature, there are probably multiple 2-tetrad monomeric G4-RNAs, stabilized by the rest of the chain.

Considering the 4-tetrad structures, only a few are observed in the one-block G4s (a total of 13). They are all G4-DNA. Except for one hybrid1 structure (6XT7 [66]), all the other 4-tetrad one-block G4s are either parallel or antiparallel.

#### Why are some topologies more prone to consist of two or three tetrads?

In a G4, the presence of several stacking layers participates in the stabilization of the structure; for the same reason, a ligand stacking on the top or the bottom tetrad stabilizes the G4 structure. However, a simple stacking of single nts on the stem does not seem sufficient to stabilize the structure; H-bonds are needed between these stacking nts to stabilize them.

In a parallel topology, when there are only two tetrads, since the loops are all propeller, and therefore at the sides of the stem, G4s tend to dimerize to maximize the number of stacking G-tetrads. This is the case of six of the thirteen 2-tetrad parallel structures; the remaining seven are monomers because of other stabilizing factors. Five of them are G4-RNA, with similar sequences and structures (5BJO, 5BJP [67], 6E80, 6E81, and 6E84 [68]), where the structure is stabilized by the stacking of 4 nts from the central propeller loop (5-nt long), which establishes Watson–Crick (WC) interactions with two 5′-FNs and two 3′-FNs (Supplementary Fig. S2A). The sixth structure, 5VHE, is stabilized by the protein, which is at the same time unfolding it, since a similar sequence, without the presence of a protein, consists of three tetrads (sixteen G4s). Finally, the seventh 2-tetrad parallel G4 is stabilized by a G-triad on its top (2N60 [69]).

All non−parallel G4-RNAs, whether they be antiparallel-basket2, hybrid2, or hybrid3, consist of 2 tetrads, stabilized by WC base-pairs (bps), in the loops or the flanking nucleotides. This is not the case with G4-DNA.

In antiparallel G4-DNA topologies, which are prone to forming two tetrads, two loops face each other at the bottom of the stem. They generally establish hydrogen bonds between their nts, which stack below the stem, stabilizing it. In addition to that, on top, there are sometimes H-bonds between the 5′-FN or 3′-FN and the central loop, which is lateral in the chair topology, and diagonal in the basket and basket2 topologies. In the two basket topologies, H-bonds are sometimes within the diagonal loop. Therefore, there is always at least one additional layer to stabilize the structure.

**Figure 2.**
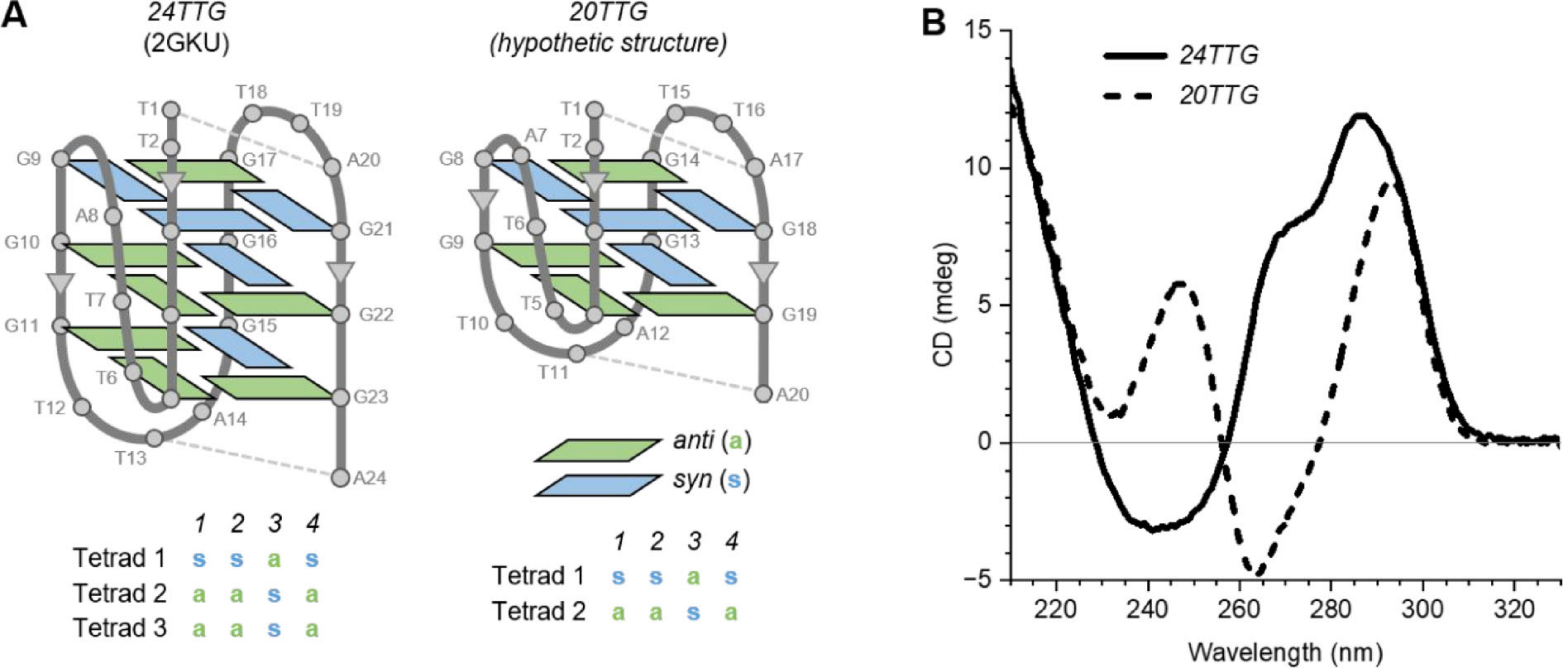
Schematic representations of *24TTG* (2GKU) and *20TTG*, and their CD spectra. (**A**) The dashed lines show the WC bps above and below the stem. Under each structure representation, the *syn* and *anti* configurations of the stem Gs are given. The numbers in italics above the gcs are the strand numbers. (**B**) CD spectra of *24TTG* and *20TTG*. Oligonucleotides were diluted from stock solutions (100 µM in H_2_O) in K100 buffer (10 mM Li cacodylate, 100 mM KCl, pH 7.2) to a strand concentration of 4 µM (total volume = 1 mL), denatured at 95°C for 5 min, cooled to room temperature, and incubated at+4°C overnight. CD spectra were recorded at 20°C in cuvettes with a path length of 5 mm.

In hybrid 1, 2, and 3 G4-DNAs, generally, the 2-tetrad structures cannot be stabilized by forming a stacking-stem dimer, like in the parallel topology, because of the presence of two lateral loops or one lateral and one diagonal loop. Besides, since the extremities of the chain end up on opposite sides (see the subsection “Relative position of the 5′ and 3′ stem extremities” below), H-bonds can only be established between an FN and a loop. This should normally stabilize the structure, but we wondered whether the H-bonds between an FN and a loop are less stabilizing than between 2 loops, like in antiparallel topologies. To check this assumption, we performed CD spectroscopy of a sequence derived from a human telomeric G4 (2GKU [70]), *24TTG*: d[TTG_3_(TTAG_3_)_3_A]. This sequence adopts a 3-tetrad hybrid1 topology stabilized by two WC bps, one on top of the stem between the first T of the 5′-FN and the A of the TTA loop 3 (L_3_), and one below the stem between the 3′-FN, A, and the second T of the TTA loop 2 (L_2_) (Fig. 2A). In principle, there is no obvious reason why these WC bps should not occur in an equivalent 2-tetrad structure. To test this, we designed a sequence where one G was deleted from each G-tract of *24TTG*, resulting in a hitherto unstudied sequence, *20TTG*: d[TTG_2_(TTAG_2_)_3_A], and compared CD spectra of both sequences in identical conditions (4 µM strand concentration, 100 mM K^+^). The CD spectrum of *24TTG* (Fig. 2B) is characteristic of a hybrid topology, as expected from the 3D structure 2GKU; it reflects the presence of a heteropolar peak (at ∼290 nm), corresponding to the stacking of tetrads 1 and 2, with their gc pattern ssas, aasa, resulting in all *syn-anti* vertical successions, and a homopolar peak (at ∼260 nm), corresponding to the stacking of tetrads 2 and 3, with their gc pattern aasa, aasa, resulting in *anti-anti* and *syn-syn* vertical successions (Fig. 2A) [13]. At 20°C, the *20TTG* sequence folds into a structure showing a CD spectrum characteristic of a G4 and presenting only a heteropolar peak. Based on the CD spectrum, we cannot exclude *a priori* an antiparallel structure; however, the heteropolar peak can also be due to the stacking of tetrads 1 and 2 in the hybrid1 topology, which corresponds to only *syn-anti* vertical successions (Fig. 2A, right panel). We favor this second hypothesis since, first, there is no intrinsic reason to transform a hybrid1 structure into an antiparallel, and second, as we will show below, if the structure were antiparallel, it could only be of the chair form because the loops are made of 3 nts, whereas there are no known antiparallel-chair htel structures in K^+^, without a mutation or a ligand. We conclude that *20TTG* adopts a hybrid1 topology, with the same stabilizing WC bps, and consequently, FN−loop H-bonds are not less stabilizing than loop-loop H-bonds. Therefore, we see no intrinsic reason why hybrid topologies should not consist of two tetrads, especially considering that three 2-tetrad hybrid structures are already present in our set. 1) 1JJP [17] (hybrid1) is an interlaced dimer. The dimerization was possible because it has two propeller loops and only one lateral loop, which is positioned at the bottom of the stem. Since the stacking-stem dimerization is always head-to-head, the bottom lateral loop does not prevent it. 2) 6JCD [63] (hybrid2) is stabilized by two layers of stacking G-Ts on top of the stem, with wobble hydrogen bonds between the snapback loop and two 3′-FNs. 3) 2MFU (hybrid3) is stabilized by a G-dyad below the stem, between the 3′-FN and the first lateral loop. Therefore, 6JCD and 2MFU are stabilized by additional layers.

In the hybrid4 topology, most structures start with a 2-nts 5′-bottom snapback. All snapbacks are found in 3-tetrad structures, except for 6JCD, which is a 2-tetrad hybrid2 G4 with a 3′-top snapback. We presume that a 1-nt 5′-bottom snapback, resulting in a 2-tetrad hybrid4 structure, would not be stable enough to form a G4. However, nothing can be said about the three hybrid4 structures without a snapback (6R9K, 6R9L [71], and 8R4E [35]) since they are also stabilized by a duplex formed by the flanking nucleotides above the stem.

Regarding the 3-tetrad antiparallel G4s, they are all G4-DNA. We hypothesize that there are few of them because of their gc succession in the strands. Indeed, as presented in [13], the vertical gc succession in antiparallel topology is heteropolar, which is *syn-anti-syn-anti* (to be read following the strand 5′ → 3′ direction, not the tetrad numbers) for a 4-tetrad G4. For a 3-tetrad G4, this succession would be *syn-anti-syn* and thus not energetically favorable, since it was demonstrated by classical and quantum-mechanical simulations that the successions ending with *syn* are less stable than those ending with *anti* [72, 73]. There are five 3-tetrad antiparallel-chair structures, all resolved in K^+^ solution, where the vertical gc succession is either of the form *syn-anti-anti* or *syn-syn-anti*. Whereas all 3-tetrad antiparallel-basket (eight structures) and basket2 (three structures) topologies either contain modified guanines or were resolved in Na^+^ solution, except for one structure, 6ZX7 [74], which has no modified guanines and was resolved in potassium. The vertical gc succession of most of these basket and basket2 topologies is of the form *syn-anti-anti*, for the first and fourth strands, and *syn-syn-anti*, for the second and third strands. So, they are always ending with *anti*-Gs, like in the chair topology. However, there are two subsets of structures (6ZX7) and (143D [40], 2MCO, and 2MCC [75]), which start with an *anti* configuration, instead of *syn* (see the subsection “Flanking nucleotides” below), and adopt the *anti-syn-anti* succession for the first and fourth strands and *syn-anti-syn* for the second and third strands. In the first subset, consisting of only 6ZX7, this is possible, probably because at the end of the G-tracts that constitute the second and third strands, there is a stacking adenine, which is *anti*, and therefore, all strands end up with an *anti*-purine. Interestingly, in a similar sequence (6ZX6 [74]), where an oxo-G with a *syn* configuration is introduced after the fourth strand, strands 1 and 4 end in a *syn*-G, but they are also followed by a stacked *anti-*A. Contrarily, for the second subset (143D, 2MCO, and 2MCC), which consists of htel G4s, with the three TTA loops, and where there are no adenines at the end of the G-tracts, two strands end up with a *syn-*G. We have no plausible explanation for why these strands would not be energetically unfavorable.

Considering the 4-tetrad structures, which are all G4-DNAs, we do not have any explanation for why they are scarce. We think that this is due to the absence of opportunity, meaning that fewer sequences with four 4G-tracts have been studied so far. When the structures of four-4G-tracts sequences are resolved in solution using NMR, they generally form 4-tetrad antiparallel-basket topologies in Na^+^ (three sequences corresponding to three structures). However, two four-4G-tracts sequences resolved in solution, corresponding to seven structures, consisted of only three tetrads: one sequence is the *RET20T* mentioned above, which adopts either 3-tetrad parallel (in K^+^) or 3-tetrad hybrid4 (in Na^+^) topologies, and one sequence from the *Tetrahymena thermophila* telomere, which adopts a 3-tetrad hybrid3 topology (in Na^+^). When the 4G-tracts G4s are resolved in a crystal using X-ray, they all form four tetrads, adopting either parallel (monomer or dimer), hybrid1, or antiparallel-basket2 topologies. We presume that the formation of four tetrads is facilitated by the crystal packing.

*To summarize*, the three tetrads in the parallel topology are required to stabilize the structure because the propeller loops generally cannot do so. Conversely, the 2-tetrad G4s are favored over the 3-tetrad G4s in antiparallel topologies for two reasons: first, because loops L_1_ and L_3_ establish stabilizing H-bonds below the stem, forming a proxy of a third layer; and second, the gc vertical succession in a 3-tetrad structure, *syn-anti-syn*, is energetically unfavorable. In the hybrid 1, 2, or 3 topologies, the presence of lateral loops prevents dimerization. The absence of 2-tetrad structures appears to be due to the lack of adequate sequences in the studies, since the H-bonds between an FN and a loop below the stem seem to be sufficiently stabilizing to form a third layer. Finally, in the hybrid4 topology, we presume that the 5′-bottom snapback should consist of 2 nts, resulting in three tetrads, to obtain a stable G4.

#### Snapbacks and bulges

In the G4-RNAs of our set, the only snapbacks are observed in the hybrid3 topology; they are all 5′-top snapbacks. Considering the bulges, all the antiparallel-basket2 structures have two bulges, and the hybrid2 structures have one bulge, whereas the hybrid3 structures have no bulges.

In the G4-DNAs, snapbacks are mostly observed in parallel and hybrid4 topologies, except for one antiparallel-chair (8PSI [76]), one hybrid1 (6XT7 [66]), and one hybrid2 (6JCD [63]) structure (Table 1). The bulges, too, are mostly observed in the parallel topology (14 structures), in addition to two hybrid3 (6AC7 [77] and 7ALU [78]) and one hybrid4 (5ZEV [15]) structure. In our set of structures, no bulges have been observed in any antiparallel G4-DNA.

### Characteristics of the two-block G4 structures

The two-block structures (Table 2 and Supplementary Table S2) consist of either four tetrads, i.e. two blocks of two G-tetrads each, or three tetrads, i.e. two G-tetrads in one block and one G-tetrad in the other block. They correspond to four main topologies observed to date: parallel/parallel, parallel/hybrid2, −/parallel, and parallel/−, where ‘−‘ stands for the absence of topology since the block consists of a single G-tetrad. However, the topology of the parallel/parallel two-block G4s is more complex than it seems since the two blocks can have the same strand directions, dddd/dddd, or opposite directions, dddd/uuuu or uuuu/dddd (each d or u corresponds to one strand direction in the block). Independent of the direction, the strand twists can be right-handed in both blocks (RH/RH), left-handed in both blocks (LH/LH), or left-handed/right-handed (LH/RH). In addition to that, for all two-block G4s, the relative twist of the blocks can also be either right- or left-handed (*RH* or *LH*). In ASC-G4, by definition, a structure that presents at least three discontinuities between the same two tetrads is given as a two-block G4. Although this is clear for the 4-tetrad structures, it can be questionable for the 3-tetrad ones. When the 3-tetrad G4 presents two different handednesses, or when its stem guanine bases (although all *anti*) have opposite relative orientations at the separation between the blocks, there is no doubt about the structure being a two-block G4. However, there exists one 49-nt-long G4-RNA sequence (the Beetroot aptamer) that is ambiguous regarding its number of blocks; it is indicated by an asterisk in Table 2 and Supplementary Table S2. The2 corresponding structures (8EYU, 8EYV, and 8EYW [79]) were resolved in the presence of different fluorescent ligands, in addition to the fourth structure (8F0N [79]), where two of the flanking nucleotides were mutated. These four structures are similar in their G4 blocks; they are given by ASC-G4 as two-block, −/parallel (−/uuuu), because of the three discontinuities between the first tetrad and the two other tetrads whose strand directions are up. However, all the twist angles between their tetrads are around 30°, corresponding to right-handed helices, like the one-block parallel structures [13]; the orientation of all stem guanine bases corresponds to the usual *anti*-Gs in parallel structures, without opposite orientations (except for the snapbacks when they exist, which are *syn*-G). Therefore, these structures can also be considered as one-block parallel G4s, with the reversed tetrad numbering, meaning that the first tetrad becomes the third one and vice versa. Consequently, the strand numbering is also reversed, meaning that strands 1→4, 2→3, 3→2, and 4→1, which results in the strand orientations dddd, with two snapbacks, 5′-bottom in strand 4 and 3′-bottom in strand 3, both in tetrad 3, and a bulge, between tetrads 2 and 3, in strand 2, accounting for the three discontinuities. In this case, they form top-to-top dimers, and their loop progression, which was +p+p+p+p−p in the two-block G4, becomes *−p*−p−p−p*+p* in the one-block G4. This is the only sequence in our set that presents such ambiguities in the topology. However, unlike parallel structures, the two extremities of the stem do not end up on opposite sides, since the two snapbacks are in the same tetrad.

**Table 2.** Characteristics of the 35 two-block structures. The slashes separate the two blocks (block1/block2). In the first column, for each topology, are given the number of structures (in bold) and the number of non-redundant sequences (in roman), the nature of the nucleotide chains (DNA or RNA), and their occurrence. The occurrence (in parentheses) is the number of structures with the corresponding characteristic. In column 2, for each block, are given the direction of the strands (d for down and u for up), the glycosidic configuration (gc) patterns (a for *anti*-G and s for *syn*-G), and the groove width signatures (w for wide, n for narrow, and i for intermediate or medium groove). When the two blocks are parallel, there is only one groove-width signature for the two blocks, but when block1 is parallel, and block2 is hybrid2, there are two groove-width signatures, one for each block. Column 3 gives the number of tetrads in the corresponding structures. Column 4 shows the handedness of each block and, in italics, the relative handedness of the two blocks. In the last column, for each topology, the loop combinations are ordered from the highest occurrence to the lowest (p for propeller, l for lateral, and *i* for internal or D-loop, + and – are the loop progression, clockwise or anticlockwise, respectively). The loop order in each combination follows the nucleotide chain sequence, and its progression is determined by looking at the G4 stem from bottom to top, whereas the order of the strand directions, the gcs, and the groove widths follows the Hoogsteen pairing, and they are determined by looking at the G4 stem from top to bottom

Topology(number of structures) (number of non-redundant sequences) nature of the chain (occurrence)	Strand directions gc pattern groove-widths	Number of tetrads	Handedness (occurrence)	Loop combinations (occurrence)
**Parallel / Parallel**(**3**) (3) DNA (3) RNA (0)	**dddd / dddd**aaaa / aaaa iiii	4	Left / *Left* / Right (2) Right / *Right* / Right (1)	−p−p−p*i+*p+p+p+*p*^a^(2) −p−p−p−p−p−p(1)
**Parallel / Parallel**(**7**) (6) DNA (**7**) (6) RNA (0)	**dddd / uuuu**aaaa / aaaa iiii	4	Left / *Left* / Left (7)	−p−p−p*i+*p+p+p+*p*(3) +p+p+p*i*−p−p−p-*p*(2) −p−p−p*i+*p+p+p+p(1)*−p*−p−p−p+*p*^b^+p+p+p+*p*(1)
**Parallel / Parallel**(**2**) (2) DNA (1) RNA (1)	**uuuu / dddd**aaaa / aaaa iiii	4	Right / *Left* / Right (1)Right / *Right* / Right (1)	−p−p−p*i+*p+p+p(2)
**Parallel / Hybrid2**(**1**) (1) DNA (1) RNA (0)	**uuuu / dddu**s^c^aaa / aaas iiii / iiwn	4	Right / *Right* / Right (1)	*−p*−p−p−p+p+p+*l* (1)
**− / Parallel**(**4**)* (2) DNA (0) RNA (**4**) (2)	**one-tetrad / uuuu**ss(sa)^d^a / aaaa iiii	3	*Right* / Right (4)	+p+p+p+p−p(4)
**− / Parallel**(**4**) (2) DNA (0) RNA (**4**) (2)	**one-tetrad / uuuu**aaaa / aaaa iiii	3	*Left* / Right (4)	−l*i+*p+p*i+*p(4)
**Parallel / −**(**12**) (8) DNA (0) RNA (**12**) (8)	**dddd / one-tetrad**aaaa / aaaa iiii	3	Right / *Right* (12)	−p−p−p(12)
**Parallel / −**(**2**) (2) DNA (0) RNA (2)	**dddd / one-tetrad**ssss / aaaa iiii	3	Right / *Left* (2)	−p*i*−p*i*−p*i*−p(2)

^a^Snapback loops are in italics: at the beginning of the combination, they correspond to 5′-snapbacks (5′-bottom or 5′-top), and at the end, to 3′-snapbacks (3′-bottom or 3′-top), *i* is for internal or D−loop.

^b^The loop in italics in the center of the combination links two different strands, one from each block (structure 6GZ6 [81]).

^c^s is due to the presence of a 3′-top snapback.

^d^These four structures are dimers. In three of them, the one-tetrad block has the gc pattern sssa in one monomer and ssaa in the other monomer, whereas in the fourth structure (8F0N [79]), the pattern in both monomers is ssaa. The gc difference is at G27 (after the bulge); it is not due to the base, which always occupies the same position as an *anti*-G, but to the sugar pucker, which is a twist (^o^_1_T) when *syn* and an envelope (^2^E, C_2’_-endo) when *anti*.

* See the discussion about this topology in the text.

#### Snapbacks and bulges

Snapbacks are only observed in some G4-DNA 4-tetrad structures (Table 2); in most parallel/parallel topologies, there is a 3′-top or 3′-bottom snapback, whereas in the parallel/hybrid2 topology, there are both a 3′-top (in block 1) and a 5′-bottom snapback (in block 2).

Unlike the one-block structures, bulges are present in many two-block structures, whether G4-RNA or G4-DNA. In the 4-tetrad structures, there are mainly between zero and two bulges, whereas in the 3-tetrad structures, the number of bulges depends on the topology: if it is of the form parallel/−, there are four bulges, all located between the first block (2 tetrads) and the second block (1 tetrad), except in one structure (5V3F [80]) where there are only three bulges because of the presence of three successive Gs in the sequence, forming the first strand; if the topology is −/parallel, there are either zero or one bulge.

### V-shaped loops are not loops

**Figure 3.**
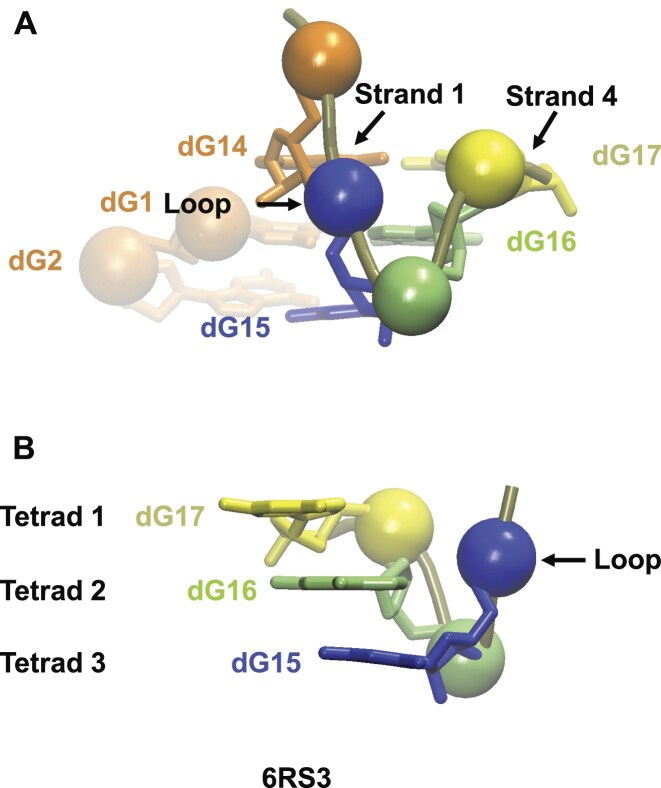
V-shaped loop of 6RS3. (**A**) Front view of strands 1 (in orange) and 4 (in blue, green, and yellow), which are connected by a 0-nt loop, i.e. the phosphate group of dG15 (its P atom is drawn as a blue hard sphere). This phosphate group is presumably the V-shaped loop. As observed, the V shape is formed by the three P atoms of the nts that form strand 4 (dG15, dG16, and dG17), and therefore, this is a V-shaped strand. (**B**) Side view of the V-shaped strand, where the guanine bases form the strand, while P atoms, or the phosphate groups, form a V-shaped backbone. The P atoms are represented by hard spheres. In (**A**), strand 1 is in transparent orange for tetrads 2 and 3 and in solid orange for tetrad 1.

V-loops are a typical feature of the hybrid4 topology, which is sometimes called a V-loop structure. Graphic programs like VMD and PyMol base their drawing of a nucleotide main chain on its phosphorus atoms. Therefore, the position of the P atoms (or, more broadly, the phosphate groups) determines the shape of the main chain. According to the most widely accepted definition of the V-shaped loop, it consists of a single phosphate group (0 nts) and adopts the shape of the letter V. This is contradictory because the V shape needs at least three points (or phosphate groups) to exist. Therefore, a 0-nt loop made of a single phosphate group cannot adopt the V shape. So, what does the V shape consist of? Fig. 3 shows a representative example of a V-shaped “loop” as a part of the hybrid4 structure 6RS3 [16]. In 6RS3, the loop that connects strands 1 and 4 consists of 0 nts, which is the phosphate group between dG14 and dG15. But, as observed in Fig. 3, the V shape is due to the phosphate groups of the three nts that constitute strand 4 (dG15, dG16, and dG17). For this reason, it seems more accurate to call this a V-shaped “strand”, which usually follows a 0-nt loop.

### The loops and their influence on topology

**Figure 4.**
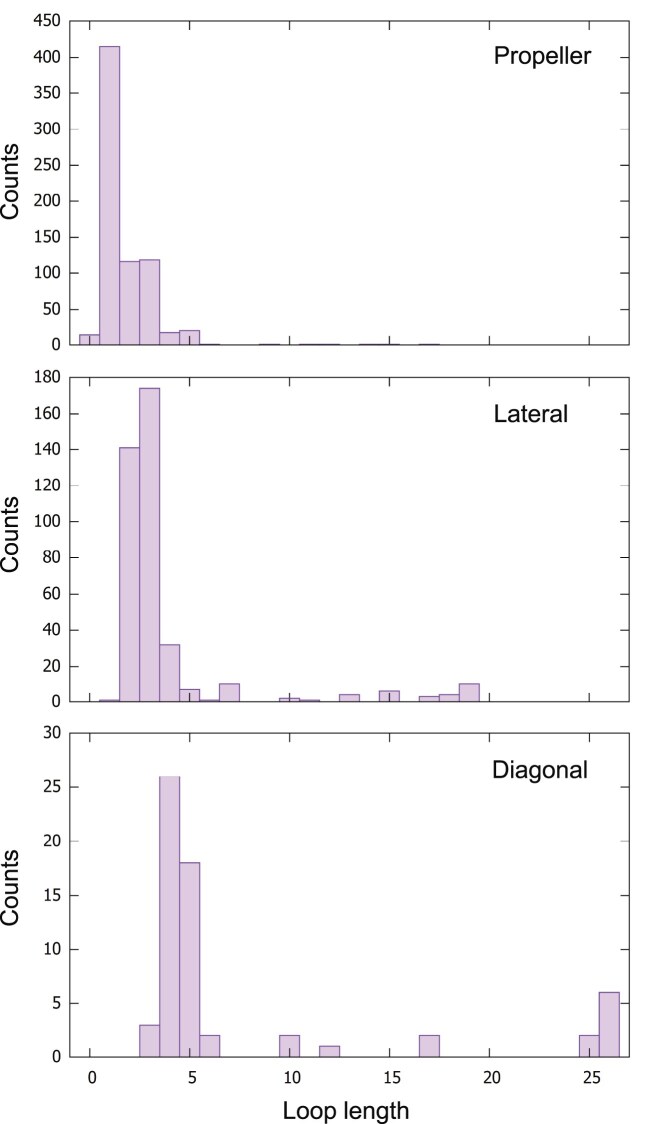
Distributions of the loop lengths for propeller, lateral, and diagonal loops. The bin size is 1 nt.

For all structures, the length of each loop (in nts) was given by ASC-G4. The distributions of these lengths, for the propeller, lateral, and diagonal loops, are shown in Fig. 4. Most propeller loops consist of 1 nt, most lateral loops of 2–3 nts, and most diagonal loops of 4–5 nts.

#### Short loops

##### Propeller loops

The 14 0-nt loops, which consist of a single phosphate group, are all propeller since they connect nts from two adjacent strands and two different tetrads. Of note, here, the strands are mostly antiparallel with snapbacks, hence the interest in broadening the definition of loops. The 0-nt loops are found in 11 of the 17 hybrid4 structures, where their progression is clockwise. The progression of the other three 0-nt loops is anticlockwise; they are found in unusual structures: the only one-block parallel structure with a D-loop (2M53), the hybrid1 interlaced dimer (1JJP [17]), and a 0-nt loop which links the two blocks of the parallel/hybrid2 structure (6KVB [18]).

Like the 0-nt loops, the 1-nt loops are all propeller, except in one structure resolved in Na^+^ solution with a 1-nt lateral loop 5J05 [31], which is antiparallel-basket. This loop links strands 3 and 4, and is probably tolerated because it immediately precedes the last G-tract.

Apart from this exception, 0- and 1-nt loops are always propeller. They can be found in all topologies: the eight one-block and the four two-block G4s. We conducted a fine analysis of their distribution in the one-block G4s, excluding 36 structures for various reasons, enumerated in the legend of Supplementary Table S3. In the remaining structures, if only L_2_, i.e. the central loop, is 1-nt long (15 G4s are in this case), the topology has 80% chance of being parallel (Supplementary Table S3). This is the highest percentage of parallel structures with only one 1-nt loop. Conversely, if only L_3_ is 1-nt long ([20] G4s), the percentage of parallel structures drops to 5%, which is the lowest. However, if L_1_ = L_3_ = 1 nt, whether L_2_ is also 1 nt ([28] G4s) or not ([48] G4s), the topology is 100% parallel. Therefore, although the number of structures is not always large enough to be affirmative, it seems that, despite the propensity of 1-nt L_2_ alone in resulting in a parallel structure, this loop is not important anymore if another loop is also 1-nt. More importantly, we observe that when any 2 loops are 1-nt long, the topology is always parallel if no stem-Gs are modified to induce the *syn* configuration. Indeed, in Supplementary Table S3, there are one G4 with L_1_ = L_2_ = 1 nt (7O1H) and three G4s with L_2_ = L_3_ = 1 nt (6R9K, 6R9L [71], and 7ZEO [82]) which are not parallel. They all contain modified guanines (^Br^G), which induce *syn* configuration. Therefore, we can conclude that when two loops are 1-nt long, the topology is parallel, whatever the length of the third loop (up to 15 nts in our set) and its composition. Now, if we look from the opposite side, meaning from that of the parallel topology, we observe that there is a difference between the structures resolved using NMR and those using X-ray crystallography. For this analysis, we exclude all the parallel structures that were excluded above (in Supplementary Table S3), that is, 22 structures. In the presence of snapbacks, we only consider the three basic loops, i.e. not snapback loops. In this subset, all one-block parallel structures resolved in solution using NMR without PEG ([83] G4s) contain at least one 1-nt propeller loop. In 72 of these NMR-resolved structures (i.e. 87%), this loop is at position 1, i.e. L_1_ = 1 nt. Similarly, in the X-ray resolved parallel structures, 25 G4s have at least one 1-nt propeller loop, and 22 of them (i.e. 88%) have L_1_ = 1 nt. However, the X-ray structures have the particularity of the presence of eight parallel G4s with L_1_ = L_2_ = L_3_ = 2 nts. Five of them are *Tetrahymena thermophila* telomeric G4s, which consist of 4 tetrads. This necessitates longer loops of 2 nts, considering the height of the stem (∼18 Å between the C5′ atoms of the extreme tetrads, compared to ∼12 Å for a 3-tetrad parallel), as demonstrated in the next paragraph. Two 3-tetrad G4s of the subset of X-ray parallel G4s with L_1_ = L_2_ = L_3_ = 2 nts, 6PNK and 6P45 [83], are short chains (18 and 20 nts, respectively) and have no equivalent sequences resolved by NMR for comparison. The remaining structure of this subset, 5VHE [84], consists of 2 tetrads and is bound to a helicase, which partially unfolds it. All the other structures with a similar sequence (16 G4s) are 3-tetrad parallel, with L_1_ = L_3_ = 1 nt, but they are either free or in complex with only small molecules. Of note, contrary to 5VHE, other sequences can form 3-tetrad G4s in the presence of a protein or a peptide. To all these NMR and X-ray parallel G4s, we can add one parallel structure resolved by electron microscopy, 8DUT [52], which consists of 3 tetrads and L_1_ = L_3_ = 1 nt.

##### Propeller loop length and the height of the stem

The average distance between two successive C5′ atoms is 6.25 Å, as calculated from the 12 221 pairs of nts in our set of 353 G4s. Then, for the 1-nt propeller loop, the distance of its C5′ atom from those of the preceding and the following guanosines should be considered, which is a total of 12.5 Å. This accommodates well the height of a 3-tetrad parallel structure (12 Å), but not that of a 4-tetrad structure (18 Å). The latter necessitates 2 nts, yielding a loop length of three times 6.25 Å, that is, 18.75 Å. The necessity of at least 2-nt loops for the 4-tetrad parallel structures was also demonstrated experimentally in [85]. This leads to a subsidiary question: if the height of a parallel 3-tetrad stem is 12 Å, how can the hybrid4 topology accommodate a 0-nt propeller loop? This is possible because the V shape of the strand that follows the 0-nt loop brings the C5′ atoms to a distance smaller than 12 Å, and the distance between the first and second nts in strand 1 is more than 6.2 Å due to the 5′-bottom snapback. For instance, in the structure 6RS3 shown in Fig. 3, the distance between the C5′ atoms of dG15 and dG17 (in tetrads 3 and 1, respectively) is 9.7 Å, and between dG1 and dG14 (i.e., the two consecutive nts in strand 1) is 7.4 Å. In the absence of the 5′-bottom snapback, there is no 0-nt loop. This is a general observation; even in non-hybrid4 topologies, the 0-nt loop only exists between two guanosines from adjacent strands and different tetrads when there is a discontinuity before one of the two nts, giving the ability to the main chain to adapt.

*To summarize*, most parallel G4s have at least one 1-nt propeller loop (mostly, L_1_), except when the G4 core is made of 4 tetrads (in which case its height necessitates 2-nt propeller loops), or when a protein starts to unfold the G4 core by diminishing its number of tetrads. However, 1-nt propeller loops are not exclusive to parallel structures; such loops can be found in all the other topologies. The 0-nt loop needs the presence of a discontinuity before one of the two guanosines it connects.

##### Lateral loops

Lateral loops are generally found in antiparallel and hybrid topologies. They are rather unusual in the one-block parallel structures, where they are all due to the presence of a snapback or a D-loop. In the two-block structures, the lateral loops are very scarce (see Table 2), and they are all made of 2 nts. An analysis based on 2-nt loops, similar to that on 1-nt loops, cannot be conducted because 2-nt loops can be either lateral or propeller, and the conclusions based only on the loop length can be distorted. Yet, the analysis of specifically 2-nt lateral loops shows that there are 73 structures with at least one 2-nt lateral loop, which adopt all topologies except hybrid4. However, the most numerous among them are those with L_1_ = L_3_ = 2-nt lateral loops (64%), in which case the structure has a good chance of being antiparallel-chair. Indeed, 45 of the 47 structures in this case are antiparallel-chair, and only 2 are antiparallel-basket (2M6W [31] and 8PSC [76]), with their central loops containing 4 or 5 nts, which is consistent with the length of diagonal loops (see below).

Since most of the lateral loops consist of 3 nts (Fig. 3), we similarly questioned the topology of the G4s that have L_1_ = L_3_ = 3-nt lateral loops. There are comparatively few. Only 10 structures have this characteristic (excluding the *22AG* structures, which are discussed below). They are all antiparallel, but their form depends on the central loop: when it is a 3-nt lateral loop, the G4 is chair (5 structures: 5YEY [86], 6GH0 [87], 6JKN [88], 7OTB [89], and 7Z9L [28]), when it is a 3-nt propeller loop, the G4 is basket2 (1 structure: 2MBJ [90]), and when it is a 4- or 5-nt diagonal loop, the G4 is either basket2 (1 structure: 2KOW [91]) or basket (3 structures: 5J4P [31], 6GZN [92], and 6ZX6 [74]).

*To summarize*, when L_1_ = L_3_ = 2–3 nt lateral loops, the topology is antiparallel-chair if L_2_ = 2–3 nts, and antiparallel-basket or basket2 if L_2_ = 4–5 nts. However, if L_1_ = L_3_ = 2–3 nts propeller loops, parallel and hybrid topologies are observed.

##### Diagonal loops

**Figure 5.**
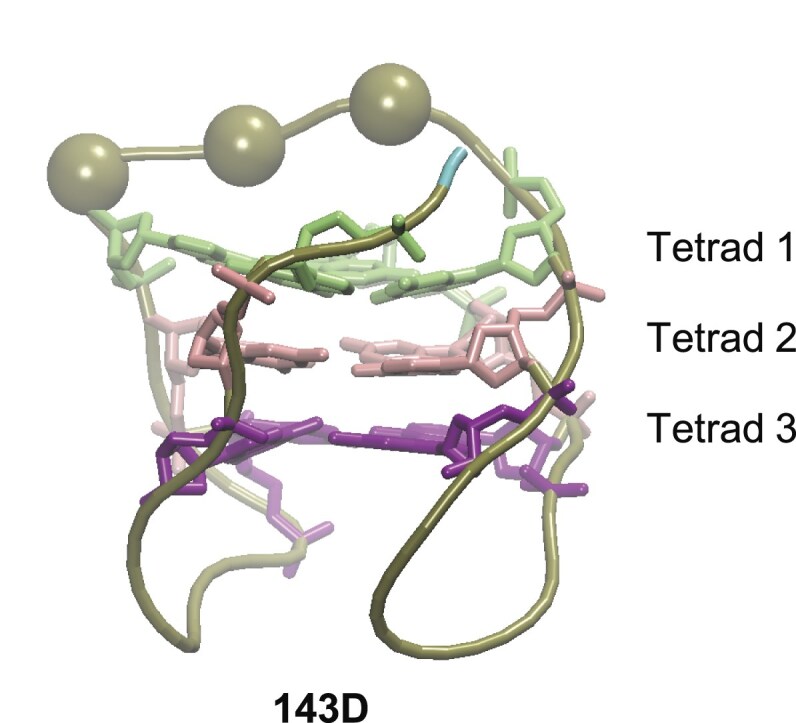
Structure 143D. The backbone is represented as a brown tube, the hydrogen atoms are omitted, and the color code is as follows: tetrad 1 in light green, tetrad 2 in pink, and tetrad 3 in purple. The P atoms of the three nts in the diagonal loop are represented as hard spheres.

There are no 1-nt or 2-nt diagonal loops, and only three 3-nt loops are found to be diagonal. They are in the three antiparallel-basket telomeric structures corresponding to the widely studied sequence, *22AG*: d[AG_3_(TTAG_3_)_3_], mentioned above: the unliganded 143D [40], which was resolved back in 1993, and the ligand-bound 2MCC and 2MCO [75], which were built upon the 143D structure and not obtained by a comprehensive analysis. They present multiple anomalies. 2MCC with eight guanines with an undetermined gc and RH/LH twists was shown to be wrong [13]. 143D has 15 sugar atoms with the wrong chirality, corresponding to 11 of the 22 nts, variably distributed across the six delivered frames. 143D and 2MCO unusually start with an *anti-*G and have two strands that end up with a *syn*-G, which is energetically unfavorable, and finally, they have a short diagonal loop of 3 nts, which, for geometrical reasons, is not compatible with a planar first tetrad. Indeed, the shortest possible loop, hypothetically straight and stretched along the diagonal of the tetrad (21.0 Å, i.e. the average distance between two diagonal C5′ atoms), needs to be raised relative to the tetrad by the van der Waals distance (3.42 Å, that is, the average rise of guanines in the stem). This results in a total minimal length of ∼28 Å (see Supplementary Fig. S3A for details). This hypothetical loop is unrealistic because it does not allow movements, but it constitutes a threshold under which the loop length is incompatible with a planar first tetrad. Now, if we consider a more realistic diagonal loop, but made of 3 nts, since the average distance between two C5′ atoms of successive nts in the sequence is 6.25 Å, the diagonal loop length will be four times this distance, resulting in a total length of 25 Å (Supplementary Fig. S3B). This is 3 Å shorter than the shortest possible loop, whereas with 4 nts, the loop’s length is over 31 Å. This allows the loop to move while maintaining the planarity of the tetrad. In 143D, the first tetrad is not planar (Fig. 5). Therefore, we conclude that the diagonal loop should be longer than three nts to take place in an unrestrained structure.

There are two G4s with two diagonal loops, the first and the third (1I34 [93] and 2MFT, unpublished); they are antiparallel-basket resolved by NMR in Na^+^. All the other diagonal loops are the central loop. They are only observed in antiparallel-basket, antiparallel-basket2, and hybrid2 topologies. In hybrid2, except for one G4-DNA structure whose loop combination is +ld−p (6L92 [94]), the basic diagonal loop, when it exists, is at the bottom of the G4, given that the stem is oriented from top (5′-tetrad) to bottom (3′-tetrad). On the contrary, in the antiparallel-basket structures, the diagonal loop is on the top of the stem. This is also the case in the antiparallel-basket2 G4-DNA structures. However, in the antiparallel-basket2 G4-RNA structures, the diagonal loop is at the bottom of the stem. These G4-RNAs have long diagonal loops of 17, 25, or 26 nts, but the difference in the diagonal loop position does not seem to be due to this length, since there is one G4-DNA antiparallel-basket structure with a long diagonal loop of 17 nts, which is positioned on the top of the stem (2M91 [95]).

*To summarize*, diagonal loops should be longer than 3 nts. Apart from snapback loops, they are only present in antiparallel-basket, basket2, and hybrid2 topologies. Their position, over or below the G4 core, depends on the topology and the type of chain (DNA or RNA). We will see below that the position of the diagonal loop has an impact on the tolerated length of the flanking nucleotides.

#### Long loops

There are seven structures with one propeller loop longer than 5 nts (up to 17 nts). One of these structures is hybrid2 (8S1W [19]), while the other six are parallel. Only in one of these parallel structures (2M92 [95]), the long propeller loop is a snapback loop; in all the others (even in the hybrid2 structure), the long propeller loop is one of the three basic loops. Thirty-three structures have lateral loops longer than 5 nts (up to 19 nts). They are observed in various topologies, including parallel, in which case these lateral loops correspond to snapbacks. A series of eight antiparallel-chair structures with more or less the same sequence has even two long lateral loops, L_1_ and L_3_, of 7 and 19 nts, respectively (8FHV, 8FHX, 8FHZ, 8FI0-2, 8FI7-8 [96]). Finally, fifteen structures have a diagonal loop longer than 5 nts (up to 26 nts). Their topologies are mostly antiparallel-basket2, but also two are antiparallel-basket, two hybrid2, and two hybrid4. In the hybrid4 topology, the diagonal loops correspond to snapbacks, whereas in the other three topologies, it is the central loop.

##### Quadruplex-duplex hybrids

In both G4-DNA and G4-RNA, the long loops (≥10 nts) are stable when they form a duplex either within the loop or between two long loops (as in the chair structures with the two loops of 7 and 19 nts, mentioned above). The minimum requirement of 10 nts corresponds to 3 WC bps plus 4 nts for the hairpin turn. However, even with 10 nts and the adequate loop composition, it might happen that only 2 WC bps form correctly, while the bp closest to the G4 core does not establish WC interactions, as in the hybrid2 structure, 5MTA [97]. This is because the duplex is in the diagonal loop (the explanation is given right below). On the other hand, the hairpin turn can be smaller than 4 nts if the loop is longer with more WC bps, as in 2M8Z [95], which is antiparallel-chair. Usually, the chair structures have their central loop made of 2–3 nts, but 2M8Z has a long central lateral loop of 15 nts, stabilized by a 6-bp duplex starting at the basis of the loop, i.e. at its extremities, contrary to 5MTA. So, the question is: why do the facing CG at the basis of the lateral loop in 2M8Z (chair) form a WC bp, while the facing TA, at the basis of the diagonal loop in 5MTA (hybrid2), do not? The explanation resides in the distance between the C5′ atoms. Indeed, in a WC bp, this distance is ∼17 Å, matching the wide-groove width in G4s [13]. Therefore, a WC bp can form between the extremities of the loop if this loop spans a wide groove. We have shown previously that the wide groove is always between two adjacent strands with down-up directions (see Fig. 5B in [13]), so, by definition, this loop is lateral since it connects two adjacent strands with opposite directions. If this loop is one of the three basic loops, the resulting topology is necessarily non-parallel, since the only topology devoid of wide grooves is the parallel one (Table 1). On the other hand, if the loop is long enough, with an adequate composition to form a duplex, while it is not attached to a wide groove, the presence of additional nts, forming bulges (not to be confounded with the bulges within the strands), is necessary to allow its adaptation. Indeed, it is necessary to increase the distance between the extremities of the loop to allow it to be diagonal, as in 5MTA, and therefore attach to stem-Gs in opposite strands, which are separated by ∼21 Å, or to shorten this distance to allow the formation of either a propeller loop, mostly on the side of the stem (∼12 Å), or a lateral loop that spans a medium (∼15 Å) or narrow (∼11 Å) groove. This is the reason why TA, at the basis of the diagonal loop in 5MTA, do not form a WC bp; T and A are here for distance adaptation to allow the formation of the other WC bps. This also explains why the presence of bulges at the junction between a duplex and the G4-stem is largely detrimental in a lateral loop, while it is stabilizing in a propeller loop [98].

*To summarize*, contrary to the claim that loop lengths have a limitation above which the G4 is destabilized (study based on only T loops [32]), the loops in G4s can be very long (≥ 10 nts), provided they establish WC interactions to form a duplex structure. This was also shown experimentally by Lim *et al*. [98]. The duplex starts at the extremities of the loop if this loop spans a wide groove; otherwise, it requires additional nts to accommodate the width of its attachment in the G4 core.

#### Loops and topology

Table 3 summarizes the relationship between the loop lengths and the topology for the one-block structures when L_1_ = L_3_, except for the 0-nt loops in hybrid4 G4s. Of the 17 hybrid4 structures, 11 have a 0-nt loop. This loop can take place either in the third or fourth G-tract, but always between strands 1 and 4. This is why it is not referred to by the loop number. Considering longer loops, in our set, when L_1_ = L_3_ = 1 nt, the topology is parallel, whatever the length of L_2_. This explains the robustness of the parallel structures mentioned in the first section, d[TGA(G_3_T)_2_A(G_3_T)_2_AA], with the loop lengths (1, 2, 1), and d[(GGA)_4_], and r[(GGA)_4_], with the loop lengths (1, 1, 1). These lengths don’t allow other topologies, under usual conditions. However, the binding of a particular ligand, such as Pt-Phen, to a structure derived from MYC (*MYC1234*) with (1, 2, 1) loop lengths, may induce a topology change from parallel (2LBY [46] and 5LIG [47]) to hybrid1, like in the two recently resolved structures, which are not in our set, 9B6Z and 8YU3 [48]. In these structures, the transformation of the 2-nt central loop from propeller to lateral, resulting in a topology change from parallel to hybrid1, is necessary to maintain the ligand on the bottom of the stem owing to the loop-ligand stacking interaction (a 2-nt propeller loop cannot stack on the ligand). This occurs at the expense of the last 1-nt propeller loop of the parallel topology, which is then replaced with the less favorable 1-nt lateral loop between strands 3 and 4, in the hybrid1 topology. In Table 1, the only hybrid1 structure with two propeller loops (1JJP) is particular, since it is an interlaced dimer. Therefore, in the absence of ligands, when the first and third loops are 1-nt long, the topology is parallel.

**Table 3.** Loop lengths of the one-block G4s and the corresponding topologies, mostly when L_1_ = L_3_. The percentage for a given combination of loop lengths is calculated as the number of structures corresponding to the topology divided by all the structures with this combination of loop lengths. For instance, when L_1_ = L_2_ = L_3_ = 2, 73% of the structures are parallel and 27% are chair. When there are several L_2_ lengths, the percentages are given for each length in the same order. If only one structure exists with the given combination, its PDB ID is provided

L_1_	L_2_	L_3_	Topology	Percentage of structures
0-nt between the strands 1 and 4 (11 structures)			hybrid4	100%
**L**_**1**_ ** = L**_**3**_ ** = 1 nt** (73 structures)				
1	1 to 15	1	parallel	100%
**L**_**1**_ ** = L**_**3**_ ** = 2 nts** (59 structures)				
2	1–2	2	parallel	100% – 73%
2	2–3 or 15	2	chair	27% – 100% or 100%
2	4–5	2	basket	100%
**L**_**1**_ ** = L**_**3**_ ** = 3 nts** (35 structures)				
3	1	3	parallel	100%
3	2	3	hybrid3	100% (6AC7 [77])
3	3	3	hybrid1hybrid3chairbasket2	50%27%19%4% (2MBJ)
3	4–5	3	basketbasket2	75%25%
**L**_**1**_ ** = L**_**3**_ ** = 4 nts** (7 structures)				
4	1 or 4	4	basket	14% (2MFT) – 43%
4	4	4	chair	14% (2KM3 [99])
4	10	4	hybrid2	29%
**L**_**1**_ ** = L**_**3**_ ** = 5 nts** (1 structure)				
5	1	5	parallel	100% (8EDP [100])
**L**_**1**_ ** ≥ 6; L**_**3**_ ** ≥ 6 nts** (8 structures)				
7	4	19	chair	100%

When L_1_ = L_3_ = 2 nts, the topology is either parallel or antiparallel, depending on the length of L_2_; then, L_1_ and L_3_ can be either propeller or lateral, respectively. For short L_2_ (1–2 nts), when the three loops are propeller, the topology is parallel, and when L_2_ = 2–3 nts and the loops are lateral, the topology is chair. This also explains the robustness of d[(G_2_T_2_G_2_)TGT(G_2_T_2_G_2_)], which is always chair since its loop lengths are (2,3,2), which doesn’t seem compatible with any other topology. For long L_2_ (≥ 4 nts), L_1_ and L_3_ are lateral while L_2_ is diagonal, and the topology is antiparallel-basket. There are two exceptions: 1) when L_2_ is very long (15 nts), the topology is chair because of the formation of a duplex within the loop, as explained above (2M8Z). 2) One 4-tetrad structure (6XT7) with L_2_ = 3 nts but where L_1_ is propeller because of the presence of a 5′-snapback and L_2_ and L_3_ are lateral loops, resulting in a hybrid1 topology. This structure is not shown in Table 3 because of the presence of a snapback, and in our set, 6XT7 is the only structure with both a 5′-snapback and L_1_ = L_3_.

When L_1_ = L_3_ = 3 nts, all topologies, apart from hybrid2 and hybrid4, are observed. In our set of one-block structures, when the 3 loops consist of 3 nts, the G4 is mostly of human telomeric sequence, and the three loops are TTA. There are three exceptions (5YEY, 7OTB, and 6GH0), where the topology is antiparallel-chair: in the first two, the loops are TTA,TTA,TTT, and in the third, they are CGA,AGG,CGT. In all (3,3,3) nt loops, we excluded those that were resolved in PEG, which are all parallel.

### Relative position of the 5′ and 3′ stem extremities

The 5′ and 3′ extremities of the G4 core can be either on the same side (i.e. in the same tetrad) or on opposite sides (in the two extreme tetrads). This information is important because, if the extremities are on the same side, adding adequate flanking nucleotides (FNs), which can form WC interactions between them, would stabilize the G4. Besides, the relative positions of the extremities would determine the orientation of the entire G4 with respect to the other strand in the genome, i.e. antiparallel or perpendicular. We therefore investigated the relative position of stem extremities in our entire set of resolved structures, whether FNs were present or not.

#### One-block G4s

In the one-block G4 structures, as expected, this relative position mainly depends on the topology, but also on the presence of some loop combinations and snapbacks (Table 4). Generally, the parallel, hybrid1, hybrid2, and hybrid3 structures end up on opposite sides, and the antiparallel structures, on the same side. The hybrid4 structures are slightly different, since most of them start with a 5′-bottom snapback, where the 5′-nt is in the middle tetrad. Therefore, although the 3′-nt is in the first tetrad, the two extremities are neither on the same side nor on opposite sides. However, three hybrid4 structures (6R9K, 6R9L [71], and 8R4E [35]) don’t have the 5′-bottom snapback, and for them, the two extremities are on the same side. Table 4 unveils some important subtleties concerning the hybrid G4-RNAs. As presented above, these structures are stabilized by WC interactions between the overhangs, so despite their hybrid topology, their loop combinations allow them to end up on the same side.

**Table 4.** Relative position of the extremities of the 318 one-block structures. The topology is given in the first column. The occurrences (in parentheses) in columns 1, 2, and 4 indicate the number of structures with the corresponding characteristic. In column 2, the information is given for most structures about the 5′ and 3′ extremities of their G4 core being located on the same side or opposite sides. In columns 3 and 4, the exceptional structures, which do not end up like the others, are reported with their snapbacks and their loop combination. ‘−’ in columns 2 and 3 indicates that at least one of the two extremities is in the middle of the G4 core

Topology (occurrence)	Side(s)	Exceptions	Loop combinations (occurrence)
Parallel (140)	**Opposite**	1 G4-DNA (6FQ2) left-handed with 5′-bottom and 3′-top snapbacks**Same**	*−p*−p−p−*p* (1)
Antiparallel-chair (64)	**Same**	1 G4-DNA (8PSI) with a 5′-bottom snapback**−**	*+l*−l−p−l (1)
Antiparallel-basket (24)	**Same**	2 G4-DNAs(1I34 and 2MFT)**Opposite**	d+pd (2)
Antiparallel-basket2 (16)	**Same**	1 G4-DNA (2MBJ)**Opposite**	+l+p+l (1)
Hybrid1 (16)	**Opposite**	No exception	
Hybrid2 (17)	RNA (6)**Same**	No exception	−p−p−l (6)
	DNA (10), DNA-RNA (1)**Opposite**	1 G4-DNA (7O1H),1 G4-DNA (6JCD) with a 3′-top snapback**Same**	−p−p−l (1),−l−p−l*d* (1)
Hybrid3 (24)	RNA (4)**Same**	No exception	*−l+*l+p+l (4)
	DNA (20)**Opposite**	No exception	−l−l−p (20)
Hybrid4 (17)	**−**	3 G4-DNA(6R9K, 6R9L, 8R4E)**Same**	+l+p+p (3)

#### Two-block G4s

Considering the two-block G4s, the structures can be formed by either 4 or 3 tetrads. As specified above, except for one structure (6KVB), the 4-tetrad G4s are parallel/parallel. However, their extremities are not all on opposite sides, as for the one-block parallel G4s. Indeed, because of the construction of these G4s in two separate blocks, the first stem-guanine or the last stem-guanine can be located in the middle tetrads. In this case, the extremities cannot be considered on any side. All the 3-tetrad parallel/− structures have their extremities on opposite sides, whereas all the 3-tetrad −/parallel structures have their extremities on the same side, including the 4 structures mentioned in the subsection “*Characteristics of the two-block G4 structures*” above, which could also be considered as one-block parallel structures (8EYU, 8EYV, 8EYW, and 8F0N). Interestingly, from this point of view, their classification in the two-block set seems justified since they end up on the same side, contrary to the one-block parallel structures. The sides of the extremities in the resolved two-block G4s are independent of the loops or the snapbacks. All these results are summarized in Table 5.

**Table 5.** Relative position of the 35 two-block structures. ‘−’ in column 2 indicates that at least one of the two extremities is in the middle of the G4 core

Topology (occurrence)	Side(s)
Parallel / Parallel (7)	**−**
Parallel / Parallel (5)	**Opposite**
Parallel / Hybrid2 (1)	**Same**
− / Parallel (8)	**Same**
Parallel / − (14)	**Opposite**

### Flanking nucleotides

#### One-block G4s

We will discuss four aspects of the overhangs in the one-block G4s: 1) the impact of 5′-FN on the glycosidic configuration of the first-stem guanine, 2) the analysis of short FNs, 3) the possibility of having long FNs in some topologies, and 4) the interactions that stabilize long FNs.

##### Impact of 5′-FN on the glycosidic configuration of the first-stem guanine

It was suggested, based on molecular dynamics (MD) [72] and quantum mechanics (QM) [73] studies, that the absence of 5′-FNs would result in a first-stem guanine *syn* configuration, because the free 5′-OH group establishes a hydrogen bond with the N3 atom of the same guanine. However, this H-bond is absent in several NMR structures of G4s lacking 5′-FN. Among the 311 one-block structures in our set (excluding the interlaced dimers), 115 have no 5′-FNs, and yet 24 of them are parallel (excluding 6FQ2 [81], which is left-handed, and 2M90 [95], which starts with a 5′-top snapback). Four of these parallel structures have an all-*syn* first tetrad (2L88 [44], 6JWD, 6JWE [5], and 8GP7 [101]). They correspond to the RET20T sequence mentioned in the “*Characteristics of the one-block G4 structures*” subsection, which adopts a hybrid4 topology in Na^+^ solution (7YS5 and 7YS7). The other 20 structures are all *anti*-G, including the first nt. Among these structures, 15 consist of a stacking-stem dimer, with a top-to-top stacking of the two parallel monomers, since dimerization stabilizes the parallel G4 despite the absence of 5′-FN [102, 103]. However, five structures resolved by NMR do not form dimers. Two of them (6YY4 [104] and 7E5P [105]) have short loops, L_1_ = L_3_ = 1 nt and L_2_ = 2 nts, which restrain the possibilities of the topology. Interestingly, a similar sequence (2LXV [106]), also resolved by NMR, but with Na^+^ instead of K^+^, adopts the same topology, but stacks as a dimer, which is probably needed, in this context, for stabilization, since it was shown that Na^+^ destabilizes the parallel topology [107] [21]. The three remaining structures (7Q48 [108], 7Q6L [108], and 7QA2), which are G4-RNAs resolved by NMR, are more problematic because, despite a short central loop (L_2_ = 1 nt), loops 1 and 3 are very long for propeller loops (L_1_ = 5 nts and L_3_ = 4 nts), especially that there are no stacking or stabilizing interactions within the loops or between the loops and the stem (Supplementary Fig. S2B). So, it is not clear why these structures adopt the parallel topology, apart from being G4-RNA, although only half of the one-block G4-RNAs, considering the non-redundant sequences, are parallel. Indeed, there is a total of 20 non-redundant one-block G4-RNA sequences. Ten of them are parallel, and 10 are non-parallel (see Table 1), but even the non-parallel G4-RNAs mostly adopt an *anti*-G configuration, by only keeping from the gc patterns the tetrads with the least *syn-*Gs. For instance, in hybrid2 G4-RNAs, the two tetrads have the gc pattern aaas, unlike the G4-DNAs, which also have an sssa tetrad (Table 1). In antiparallel-basket2, the first tetrad has the configuration pattern aasa, while the second is the usual aass. There is an exception with the hybrid3 G4-RNA sequence, which is all *anti-*G, conferring the 3D-structure some additional characteristics from parallel and antiparallel-basket2 topologies, also. This is why this G4-RNA was called platypus in reference [13]. All G4-RNA structures have their first stem-guanine with an *anti* configuration, whether their topology is parallel or not.

The parallel topology is determined by the presence of 1-nt loops, whether 5′-FN is present or not. In the absence of 5′-FN, the first tetrad is either all *syn-*G or a dimerization is needed. However, in regular G4-DNA of non-parallel topologies (155 G4s), structures start with a *syn*-G, except in a few cases (10 G4s, of which three are the structures with anomalies, five have modified guanosines, one starts with a 5′-bottom snapback, and 6ZX7, with its *anti*-A’s ending strands 2 and 3, mentioned above). In the absence of 5′-FN (86 G4s), the usual explanation for the *syn*-configuration of the first-stem G is the formation of an H-bond between its N3 and the free 5′-OH group (despite the absence of this H-bond in most NMR structures). However, what is the rationale for adopting this configuration in the presence of 5′-FN (69 G4s), given that *syn*-dG was shown to be less energetically favorable than *anti*-dG by QM calculations [109]? The reason is the presence of one or two upward strands in hybrid or antiparallel topologies, respectively. In the upward strands, guanines must adopt reversed configurations relative to the downward strands to insert into the Hoogsteen H-bond network. But, since ending a strand with a *syn*-G is highly unfavorable, the upward strands end up with *anti*-Gs located in the first tetrad; consequently, the guanine of the downward strand located in this tetrad should adopt a *syn* configuration to fulfill the Hoogsteen interactions. Hence, the first-stem guanine adopts a *syn* configuration as soon as there is an upward strand in the structure, leading to either antiparallel or hybrid topologies. This reason is general, even in the absence of 5′-FN. Importantly, this suggests that the folding of the G4 does not start from its 5′-end, but rather from its loops. When the loop length is more favorable to form a lateral loop, the two adjacent strands are in opposite directions, necessitating the presence of *syn* configurations.

##### Analysis of short FNs

In the one-block G4s, excluding the interlaced dimers, all topologies exist in the absence of only 5′-FNs, only 3′-FNs, or both. Similarly, when only short FNs are considered (1–4 nts), 153 structures with short 5′-FN and 123 short 3′-FN also adopt all topologies (naturally, most of these structures are counted twice when they have both short 5′ and 3′ FNs). Particularly, when 5′-FN = 3′-FN = 1 nt, FNs were observed to form a WC bp, only in one chair topology (7CV4 [110]), where this bp is on top of the G4 stem and spans the first wide groove. In this conformation, loop 2 forms a duplex, also on top of the stem, spanning groove 3, which is also wide (Supplementary Fig. S4A). Interestingly, when two nts are added to the 5′-extremity and the 3′-FN is removed from this sequence, the topology is modified to hybrid1 (7CV3 [110]), while the L_2_ duplex is maintained, but below the stem, also spanning the wide groove (Supplementary Fig. S4B). Considering the case of 5′-FN = 3′-FN = 4 nts, it was only found in the three hybrid4 structures (6R9K, 6R9L, 8R4E), lacking the 5′-bottom snapback. In this case, 5′-FN and 3′-FN form together 4 WC bps, also spanning the wide groove (Supplementary Fig. S4C). Interestingly, when in the sequence of 8R4E, the FNs are modified in number (3 nts) and composition (5′-FN: CGCT → TGA, 3′-FN: ACGC → TAA), the topology is changed to hybrid3 (8R4W [35]) because a WC bp between the two middle mutant nts, G and A, cannot be established (Supplementary Fig. S4D).

##### Possibility of having long FNs in some topologies

For longer FNs (more than 4 nts), some topologies are not observed. Indeed, none of the 42 structures with long 5′-FNs or 46 with long 3′-FNs adopt antiparallel-basket, hybrid1, or hybrid4 topologies. We investigated whether this is due to an inherent reason or just to the absence of opportunity, meaning that no one tried to resolve such structures. Long FNs can destabilize the structure if they do not establish WC interactions because of their increased entropy and kinetic energy; if this energy is transmitted to the stem, it weakens its Hoogesteen interactions. Considering the hybrid4 topology, the absence of long FNs is normal when the structure starts with a 5′-bottom snapback, as in the majority of these G4s. However, in the three hybrid4 structures (6R9K, 6R9L, 8R4E), which lack this snapback, the absence of long FNs is due to the absence of opportunity, since when 5′-FN and 3′-FN are 4-nt long, they form WC bps on the top of the stem (Supplementary Fig. S4C); therefore, elongating them with adequate nts, prone to forming WC bps, seems possible. Regarding the hybrid1 structures, they are all G4-DNAs, and 87% are telomeric. The two stem extremities end up on opposite sides of the stem (Supplementary Fig. S5A), and therefore, to establish WC bps with the 5′-FN, the 3′-FN should be long enough to turn and overcome the presence of the lateral loop on the top of the stem. This seems improbable, although a similar scenario takes place in 5 parallel structures, from two close sequences, where 5′-FN and 3′-FN, ending up also on opposite sides, have long FNs which form duplexes on the top side of the stem (Supplementary Fig. S2A). However, these parallel structures have important differences; they are G4-RNAs, consist of only 2 tetrads, and the first 3′-FN does not establish a WC interaction with a facing nt below the stem, like in the hybrid1 structures. Finally, considering the antiparallel-basket structures, they are also all G4-DNA, but the stem extremities end up on the same side, i.e. on the top of the stem. Here, the difficulty for the formation of a duplex between the FNs is due to the systematic presence of the diagonal loop on the top of the stem (Supplementary Fig. S5B). Probably, long FNs would be possible if, between the putative duplex and the stem extremities, there were enough nts to go beyond the diagonal loop and preferentially interact with it. Similarly, in the G4-DNA basket2 structures, the diagonal loop is located on the top of the stem, and the FNs are short as well. However, in the G4-RNA basket2 structures, the diagonal loop is situated at the bottom side of the G4-stem, and long FNs, forming an RNA duplex, are observed on the top of the stem.

##### The interactions that stabilize long FNs

Regarding the long FNs, there are several ways to stabilize them. Generally, 5′-FN and 3′-FN are about the same length to be able to constitute a duplex. However, in one parallel G4-DNA, resolved using electron microscopy (8DUT [52]), the 5′- and 3′-FNs do not interact, despite being of the same length (15 nts each), but rather both hybridize to an additional 46 nt−long nucleotide chain (Supplementary Fig. S6A). On the contrary, in some G4-RNA hybrid2 structures, like 6E8S [111], although the 3′-FN is 5-nt longer than the 5′-FN, it hybridizes with the latter after making a short hairpin (Supplementary Fig. S6B). In some other cases, where the lengths of 5′-FN and 3′-FNs are very different (Δ*n* from 4 nts to 17 nts in our set), the overhangs do not interact with each other. This interaction is substituted with several possibilities: (1) a WC interaction of 3′-FN with the central lateral loop, like in a chair topology (6GH0) (Supplementary Fig. S6C); (2) the formation of a hairpin within the long FN alone (like in the parallel 6ZL2) (Supplementary Fig. S6D); (3) the interaction of the long FN with a protein (like in the parallel 5VHE) (Supplementary Fig. S6E); or (4) the formation of a duplex between the long FN and a short additional nucleotide chain (like in the parallel 5DWW [112]) (Supplementary Fig. S6F).

#### Two-block G4s

In the two-block G4s, in only two topologies we observed the presence of 0- to 1-nt 5′-FN and 0- to 2-nt 3′-FN: 1) in all the 4-tetrad structures, where each block consists of two tetrads, whatever the direction of the strands, and 2) in the two RNA 3-tetrad structures (7MKT [113] and 8TNS [114]), with the topology parallel/−, where the first block (2 tetrads) is completely formed by *syn*-Gs.

In all the other 3-tetrad structures, 5′-FN and 3′-FNs are long and form an RNA duplex. In the −/parallel topologies, whose extremities are on the same side, the duplex is formed between 5′-FN and 3′-FN of the same chain. If one of the FNs is slightly longer than the other, it forms an additional loop (like in 8EYU) (Supplementary Fig. S7A) or a short duplex with the basic loops (like in 5DEA [115]) (Supplementary Fig. S7B). However, in the parallel/− topologies, where the extremities are on opposite sides, either the RNA duplex is also between 5′- and 3′-FNs of the same chain (like in 5V3F [115]), where the duplex is on the side of the stem with its main axis perpendicular to that of the G4-stem (Supplementary Fig. S7C), or the duplex forms between the 5′-FNs from different chains (like in 6V9B [116]) (Supplementary Fig. S7D). This shows the variability of RNA duplexes in the two-block 3-tetrad G4s.

## Conclusions

This comprehensive study, based on the analysis of 353 intramolecular structures, allowed us to clarify some information in the literature and shows that, although G4 structures are much more complex than could be expected considering their small size, one can observe some rules that seem to govern their fold.

### Literature clarification

1- Parallel and hybrid G4s are mostly made of 3 tetrads, the former because they don’t have lateral loops to establish the interactions that would sufficiently stabilize a 2-tetrad structure, and the latter for undetermined reasons. Conversely, antiparallel structures are mostly made of 2 tetrads, because in 3-tetrad structures, the heteropolar vertical stacking of guanines would end up with the unfavorable *syn*-G configuration.
2- In our set of G4-DNA structures, most snapbacks are in parallel and hybrid4 topologies, and most bulges are in parallel topology. Contrarily, the antiparallel topologies are devoid of bulges, and only one antiparallel-chair has a snapback.
3- In the literature, hybrid2 and hybrid3 topologies are both referred to as “hybrid2” because the strand with the direction up (u) opposite to the three others is adjacent to the first strand. Here, we show that, if the u-strand is to the left of strand 1 (hybrid3), the structural characteristics are very different from when it is to its right (hybrid2). Indeed, except for two structures, all hybrid2 G4-DNAs contain one diagonal loop, one propeller loop, and one lateral loop, whereas all hybrid3 are devoid of diagonal loops; they contain one propeller and two lateral loops. Hybrid2 G4-RNAs are also devoid of diagonal loops, but they contain two propeller loops and one lateral loop. Hence, the importance of distinguishing between hybrid2 and hybrid3 topologies.
4- Contrary to a common idea, most resolved one-block G4-RNAs consist of two tetrads, which are stabilized either by an RNA duplex above and/or below the G4-stem for long chains, or by dimerization for short chains.
5- The V-shaped loops are, in fact, V-shaped strands, which most typically follow 0-nt loops, and 0-nt loops need the presence of a discontinuity before one of the two nts they connect.

### Role of the loops in the G4 topology

6- The 0- and 1-nt loops are always propeller, except when an external strain (Na^+^ or a ligand) forces the 1-nt loop 3 to become lateral. Most of the lateral loops are made of either 2 or 3 nts. All diagonal loops, even snapbacks, should be ≥ 4 nts. The 3-nt diagonal loops that were observed in 143D, 2MCO, and 2MCC result in a non−planar tetrad. The diagonal loops, apart from snapbacks, are only observed in antiparallel-basket, antiparallel-basket2, and hybrid2 topologies. In G4-DNA basket and basket2, the diagonal loop is situated above the stem, whereas in G4-RNA basket2 and most G4-DNA hybrid2, it is below.
7- The presence of three 1-nt loops necessarily results in three propeller loops and therefore a parallel topology. When only two loops are 1-nt (propeller), in the absence of strain due to a ligand (like Pt−phen) or to guanine modification (like ^Br^G, which induces a *syn* configuration), the topology is always parallel, independently of the length of the third loop. However, when only one loop is 1-nt long, the probability of adopting a parallel topology is the highest (87%) when this loop is L_2_.
8- When L_1_ = L_3_ = 2 nts, the topology depends on the length of L_2_: if L_2_ = 1 nt, the topology is parallel; if L_2_ = 2 nts, it is mostly parallel (73%), but can also be antiparallel-chair; if L_2_ = 3 nts or L_2_ is long enough with a nucleotide sequence that allows it to be structured in a duplex, the topology is also antiparallel-chair; whereas, if L_2_ = 4–5 nts, the topology is antiparallel-basket. Expectedly, the longer the loops, the more variability is observed in the topology. Warily, the number of nts separating the G-tracts should not be confused with the number of nts in the loops because some Gs of the G-tracts can be parts of these loops.

### Role of geometry in the G4 folding

9- The average distance between the C5′ atoms of two successive nucleotides is 6.25 Å. This determines the geometry of the G4: a 4-tetrad parallel G4 needs at least 2-nt propeller loops, and a diagonal loop should consist of at least 4 nts.
10- When a loop forms a duplex, in the absence of a bulge between the duplex and the stem, i.e. if it directly follows (or precedes) a stem-guanine on each extremity, the basis of the loop width is about 17 Å, resulting in a lateral loop which spans a wide groove. Contrarily, in the presence of intermediate nts (bulges), the basis of the loop width can adapt to be either larger (about 21 Å) resulting in a diagonal loop or shorter, to form a lateral loop which spans a medium (∼15 Å) or narrow (∼11 Å) groove, or a propeller loop, mostly on the side of the stem (∼12 Å for a 3-tetrad structure).

### Role of overhangs in the G4 stabilization

11- A G4 can be stabilized by overhangs that form a duplex on the top or the bottom side of the G4. In this case, it is important to know whether the stem extremities are located on the same side or on opposite sides of the G4 stem. We show that, apart from a few exceptions, parallel and hybrid1 topologies end up on opposite sides, whereas antiparallel (chair, basket, and basket2) and hybrid4 (in the absence of 5′-bottom snapback) topologies end up on the same side. In hybrid2 and hybrid3 topologies, the extremities are on opposite sides for G4-DNAs and on the same side for G4-RNAs.
12- In the two-block structures, the parallel/parallel topologies end up on opposite sides when at least one of the extremities is not in the middle of the stem. In the −/parallel structures, the extremities end up on the same side, whereas in the parallel/− structures, they end up on opposite sides.
13- The absence of 5′-FN usually results in non-parallel G4 topologies, since it favors a *syn* configuration for the first-stem guanine. However, this absence may allow the parallel topology, provided that either the stem starts with an all-*syn* tetrad or the G4 forms a stacking-stem dimer.
14- In the presence of 5′-FN, the vast majority of non-parallel G4-DNA have their first stem-guanine in a *syn* configuration. Apart from one plausible case, the few exceptions are due to modified guanosines or the presence of a 5′-snapback. The first-stem guanine adopts a *syn* configuration, despite its less favorable energy compared to *anti*-G, because of the presence of upward strands. This suggests that the G4 folding does not start from the 5′-end of the stem but rather from the loops.
15- Long overhangs stabilize the structure provided they are buried within a protein or establish WC interactions between each other, with the loops, or with additional nucleotide chains. The WC interactions between 5′-FNs and 3′-FNs are possible, even if less probable, when the extremities of the stem end up on opposite sides, as in parallel one-block or two-block topologies. However, it seems difficult to obtain a duplex on top of an antiparallel-basket or basket2 G4-DNA because, in this case, the diagonal loop is also located on the top of the stem, preventing the 5′-FNs and 3′-FNs from coming in close contact, unless they are long enough to overtake this loop. In antiparallel-basket2 G4-RNAs, the diagonal loop is at the bottom of the stem, and RNA duplexes, made of 5′-FNs and 3′-FNs, were observed on the top of these structures.

These rules may help in designing and resolving some new G4 structures.

## Supplementary Material

gkag435_Supplemental_Files

## Data Availability

Data used for these analyses are gathered in an Excel file added to the Supplementary Data, titled “Sequences_and_characteristics.xlsx.”
