# Supplementary material for: Topological rules and anomalies in intramolecular G-quadruplex folding: a comprehensive study
Source: Nucleic Acids Res. 2026 May 19;54(9):gkag435. doi: 10.1093/nar/gkag435 (PMC13183678; doi:10.1093/nar/gkag435)
Supplement: gkag435_Supplemental_Files [file gkag435_supplemental_files.zip › Supplementary_Data.pdf]

SUPPLEMENTARY DATA

Topological Rules and Anomalies in Intramolecular G-Quadruplex Folding: A Comprehensive Study

Anton Granzhan and Liliane Mouawad *

CONTENTS

p. 2	Figure S1. Schematic representation of the intramolecular G4 discontinuities.
p. 3	Legend of the Sequences-and-characteristics.xlsx file.
p. 4	Table S1. Topology and PDB IDs of the 318 one-block structures.
p. 5	Table S2. Characteristics of the 35 two-block structures.
p. 6	Figure S2. Structures of two parallel G4-RNAs.
p. 7	Table S3. Number of parallel structures when at least one loop consists of 1 nt.
p. 8	Figure S3. Demonstration for the need for more than 3 nts to form a diagonal loop.
p. 9	Figure S4. Small modifications in short FNs can produce important conformational changes.
p. 10	Figure S5. Exploration of the possible presence of long FNs.
p. 11	Figure S6. Various ways for stabilizing long FNs in the one-block G4s.
p. 12	Figure S7. Various ways for stabilizing long FNs in the 3-tetrad two-block G4-RNAs.

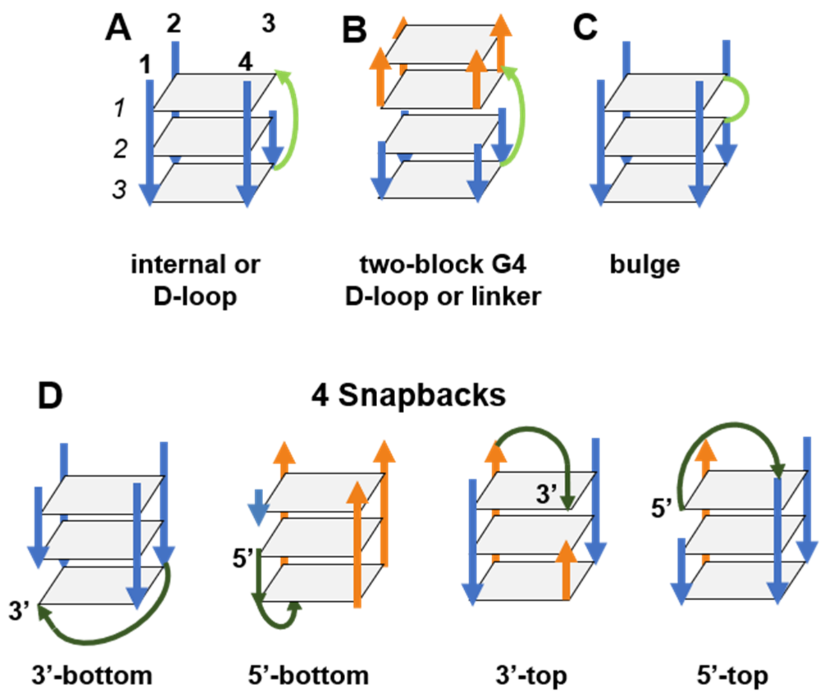

Figure S1. Schematic representation of the intramolecular G4 discontinuities. The internal or D-loop in a one-block G4 (A), the linker between the two blocks, which is also a D-loop in a two-block G4 (B), a bulge (C), and the four types of snapbacks (D). (A-D) The tetrads are drawn as light gray planes, the strands as arrows, blue for down and orange for up. The propeller, lateral, and diagonal loops are omitted for clarity. (A-C) The D-loops and the bulge are in green. (A) The numbers in bold at the top of the stem are those of the strands, and the slanted numbers to the left are those of the tetrads. (D) In the four types of snapbacks, the 5' and 3' extremities of the stem, which snap back, are indicated, and the snapback loops are in dark green.

Legend of the Sequences-and-characteristics.xlsx file:

This file contains all the 353 sequences used in this study, and their colored counterpart, where Gs are colored according to the strand they belong to: **Strands 1 2 3 4**. In the colored sequences, the modified nts are designated by the letter M, only when the keyword “MODIFIED RESIDUES” in the PDB file is filled, otherwise it is omitted, whereas in the black sequences, they are always given. The modified nts are reported in the “Modifications” column, where they are enumerated in the order of their appearance in the sequence, using their abbreviations from the PDB file. The chemical name of these modifications is in the sheet titled “mods”. In column 1, there are the PDB IDs, in column 2, the nature of the G4, DNA, RNA, or hyb (meaning hybrid DNA-RNA), and in column 3, the number of nts in the chain. In the “Topology”, the ‘/’ separates block1 / block2 in a two-block G4. When a topology is attributed to a block, it means that the block consists of two G-tetrads, whereas when ‘−‘ is attributed to the block, this block consists of only one tetrad. The “Small-molecule Ligand” and “Protein-Peptide” columns indicate the number of ligands or proteins attached to the nucleotide chain. “Expdta” is the experiment used to resolve the structure. The “#Tetrads” column gives the number of G-tetrads in each G4 monomer. In this column, 2-3 means that ASC-G4 detected two G-tetrads, whereas the third tetrad consists of 3 Gs supplemented with a G-mimicking molecule, which is given in the Remarks column, starting with a ‘+’. In the “Dimerization” column, ‘dimer’ only designates a stacking-stem dimer, while ‘hid-dimer’ stands for a hidden stacking-stack dimer. This case is only encountered in crystal structures, where there is a dimer, but in the Asymmetric Unit Cell (AUC), and therefore, in the PDB file, there is only one monomer. The existence of a dimer is hinted at by the presence of an extra cation located at the surface of the monomer, on its central axis. In the “#Blocks” column, the number of blocks is given, 1 for one-block G4s, 2 for two-block G4s, and 0 for the interlaced dimers, where the first guanine of a monomer inserts in the first tetrad of the other monomer and vice versa. In the “Handedness” column, for the two-block G4s, there is the handedness of each block, except when the block consists of one tetrad, and the handedness of one block relative to the other (see Table 2 in the main text). “Loops-combination” is the same as those reported in Tables 1 and 2 of the main text, without the indication about the snapbacks, which can be deduced from the colored sequences, when a single G, far from the others, is colored.

Table S1. Topology and PDB IDs of the 318 one-block structures. In the first column are given the topology, the nature of the nucleotide chains (DNA or RNA) and their number (in parentheses), the direction of the strands (d for down and u for up), in parentheses the main glycosidic configuration (gc) patterns (a for *anti*-G and s for *syn*-G), and the groove width signatures (w for wide, n, for narrow and i for intermediate or medium groove). #Struc. Refers to the number of structures. The PDB IDs of G4-RNAs are in bold, and that of the hybrid G4-DNA-RNA is in slanted bold.

Topology	#Struc	PDB ID
**Parallel**<br>DNA (125), RNA (15)<br>dddd,(aaaa,ssss),iiii	140	1KF1, **1MY9**, 1MYQ, 1XAV, 1Y8D, 2A5P, 2A5R, 2KQG, 2KQH, 2KYP, 2KZE, 2L7V, 2L88, 2LBY, 2LD8, 2LE6, 2LEE, 2LK7, 2LPW, 2LXQ, 2LXV, 2M27, 2M4P, 2M53, 2M90, 2M92, 2M93, 2MB2, 2MB4, 2MGN, 2N21, 2N4Y, 2N60, 2N6C, 2O3M, **2RQJ**, **2RSK**, **2RU7**, 3CDM, 3QXR, 3R6R, 3SC8, 3T5E, 3UYH, 4DA3, 4DAQ, 4FXM, 4G0F, 4WO2, 4WO3, **5BJO**, **5BJP**, 5CCW, 5DWW, 5DWX, 5I2V, 5LIG, 5NYS, 5NYT, 5NYU, 5UA3, 5VHE, 5W77, 6AU4, **6E80**, **6E81**, **6E84**, 6ERL, 6FQ2, 6H5R, 6IP3, 6IP7, 6ISW, 6JJ0, 6JWD, 6JWE, 6K3X, 6K3Y, 6LDM, 6LNZ, 6N65, 6NEB, 6O2L, 6P45, 6PNK, 6Q6R, 6SUU, 6T2G, 6T51, 6V0L, 6W9P, 6WCK, 6XCL, 6YY4, 6ZL2, 6ZL9, 6ZRM, 6ZTE, 7CLS,7E5P, 7JKU, 7KBV, 7KBW, 7KLP, 7LL0, 7MSV, 7N7D, 7N7E, 7NWD, 7OAR, 7PNE, 7PNG, 7PNL, **7PS8**, **7Q48**, **7Q6L**, **7QA2**, 7QVQ, **7SXP**, 7WGW, 7X7G, 7X8M, 7X8N, 7X8O, 7XDH, 7XH9, 7XHD, 7XIE, 7ZEM, 8ABD, 8D78, 8D79, 8DUT, 8EBO, 8EDP, 8GP7, 8JFQ, 8PSB, **8Q4O**, 8X1V
**Antiparallel-chair**<br>DNA (64), RNA (0)<br>dudu,(sasa,asas),wnwn	64	148D, 1BUB, 1C32, 1C34, 1C35, 1C38, 1HAO, 1HAP, 1HUT, 1QDF, 1QDH, 1RDE, 2IDN, 2KM3, 2LYG, 2M8Z, 2N2D, 3QLP, 4DIH, 4DII, 4LZ1, 4LZ4, 4NI7, 4NI9, 5CMX, 5EW1, 5EW2, 5MJX, 5OPH, 5YEY, 6EO6, 6EO7, 6EVV, 6FC9, 6GH0, 6GN7, 6JKN, 6Z8V, 6Z8W, 6Z8X, 7CV4, 7D31, 7D32, 7D33, 7NTU, 7OTB, 7V3T, 7W9N, 7Z9L, 7ZKL, 7ZKM, 7ZKN, 7ZKO, 8ABN, 8BW5, 8FHV, 8FHX, 8FHZ, 8FI0, 8FI1, 8FI2, 8FI3, 8FI8, 8PSI
**Antiparallel-basket**<br>DNA (24), RNA(0)<br>duud,(saas,assa),wini	24	143D, 1I34, 201D, 230D, 2KF7, 2KF8, 2KKA, 2M6V, 2M6W, 2M91, 2MCC, 2MCO, 2MFT, 5J05, 5J4P, 5J4W, 5J6U, 5LQG, 6GZN, 6ZX6, 6ZX7, 7OQT, 8JIC, 8PSC
**Antiparallel-basket2**<br>DNA (7), RNA (9)<br>dduu,(ssaa,aass),iwin, iwii	16	2KOW, 2MBJ, **4KZD**, **4KZE**, **4Q9Q**, **4Q9R**, 5LQH, **5OB3**, **6B14**, **6B3K**, 6F4Z, 6FTU, 6YEP, **7L0Z**, **7ZJ4**, 8JIH
**Hybrid1**<br>DNA (16), RNA (0)<br>ddud,(ssas,aasa),iwni	16	1JJP[I], 2E4I, 2GKU, 2HY9, 2JSK, 2JSM, 2MAY, 2MB3, 2MWZ, 5MBR, 5Z80, 5Z8F, 6IA4, 6KFI, 6XT7, 7CV3
**Hybrid2**<br>DNA (10), RNA (6), DNA-RNA (1)<br>dddu,(sssa,aaas),iiwn	17	2LOD, 5MCR, 5MTA, 5MTG, 5OV2, **6E8S**, **6E8T**, **6E8U**, ***6FFR***, 6JCD, 6L92, **6PQ7**, **6UP0**, 7OLH, 8PSE, 8S1W, **8U5J**
**Hybrid3**<br>DNA (20), RNA (4)<br>dudd,(sass,asaa),wnii	24	186D, 2F8U, 2JPZ, 2JSL, 2JSQ, 2KZD, 2MFU, 5MVB, 6AC7, 6CCW, 6IA0, 6KFJ, 7ALU, 7EL7, **7OA3**, **7OAV**, **7OAW**, **7OAX**, 7X2Z, 7X3A, 8IJC, 8R4W, 8R6D, 8R6H
**Hybrid4**<br>DNA (17), RNA (0)<br>duuu,(saaa,asss),wiin	17	2KPR, 5O4D, 5ZEV, 6H1K, 6L8M, 6R9K, 6R9L, 6RS3, 6TC8, 6TCG, 6YCV, 7ATZ, 7YS5, 7YS7, 7ZEK, 7ZEO, 8R4E

Table S2. Characteristics of the 35 two-block structures. The slashes separate the two blocks (block1 / block2). In the first column are given the topology, the nature of the nucleotide chain (DNA or RNA), then, for each block, the orientation of the strands (d for down and u for up) and the main glycosidic configuration (gc) patterns (a for *anti*-G and s for *syn*-G), and finally, the groove width signatures (w for wide, n, for narrow and i for intermediate or medium groove). The occurrence (in parentheses) is the number of structures with the corresponding characteristic. Column 2 gives the loop combinations ordered from the highest occurrence to the lowest (p for propeller, l for lateral, and *i* for the linkers between the blocks, + and – are the loop progression, clockwise or anticlockwise, respectively). The loop order in each combination follows the nucleotide chain sequence, and its progression is determined by looking at the G4 stem from bottom to top, whereas the order of the strands, the gcs, and the groove widths follows the Hoogsteen pairing, and they are determined by looking at the G4 stem from top to bottom. In the last column, the PDB ID corresponding to each loop combination is given. The PDB IDs of G4-RNAs are in bold.

**Topology (occurrence)**	**Loop combinations (occurrence)**	**PDB ID**
Parallel / Parallel (3)<br>DNA (3), RNA (0)<br>dddd / dddd<br>aaaa / aaaa (iiii)	-p-p-p*i*+p+p+p+*p*[1] (2)<br>-p-p-p-p-p-p (1)	6JCE, 6QJO<br>2N3M
Parallel / Parallel (7)<br>DNA (7), RNA (0)<br>dddd / uuuu<br>aaaa / aaaa (iiii)	-p-p-p*i*+p+p+p+*p* (3)<br>+p+p+p*i*-p-p-p-*p* (2)<br>-p-p-p*i*+p+p+p+p (1)<br>-*p*-p-p-p+*p*[2]+p+p+p+*p* (1)	7D5D, 7D5E, 7D5F<br>2MS9, 4U5M<br>7DFY<br>6GZ6
Parallel / Parallel (2)<br>DNA (1), RNA (1)<br>uuuu / dddd<br>aaaa / aaaa (iiii)	-p-p-p*i*+p+p+p (2)	1OZ8, **6K84**
Parallel / Hybrid2 (1)<br>DNA (1), RNA (0)<br>uuuu / dddu<br>s[3]aaa / aaas (iiii / iiwn)	-*p*-p-p-p+p+p+*l* (1)	6KVB
− / Parallel (4)*<br>DNA (0), RNA (4)<br>one-tetrad / uuuu<br>ss(sa)[4]a / aaaa (iiii)	+p+p+p+p-p (4)	**8EYU, 8EYV, 8EYW, 8F0N**
− / Parallel (4)<br>DNA (0), RNA (4)<br>one-tetrad / uuuu<br>aaaa / aaaa (iiii)	-l*i*+p+p*i*+p (4)	**2LA5, 5DE5, 5DE8, 5DEA**
Parallel / − (12)<br>DNA (0), RNA (12)<br>dddd / one-tetrad<br>aaaa / aaaa (iiii)	-p-p-p (12)	**5V3F, 6C63, 6C64, 6C65, 6V9B, 6V9D, 8U5K, 8U5P, 8U5R, 8U5T, 8U5Z, 8U60**
Parallel / − (2)<br>DNA (0), RNA (2)<br>dddd / one-tetrad<br>ssss / aaaa (iiii)	-p*i*-p*i*-p*i*-p (2)	**7MKT, 8TNS**

[1] Loops in italics are due to the presence of a snapback: at the beginning of the combination, it is a 5’-snapback, and at the end, it is a 3’-snapback
[2] The loop in italics in the center of the combination links two strands, one from each block
[3] s is due to the presence of a 3’-top snapback
[4] These structures are all dimers. In 8F0N, the gcs of the first block in both chains are ssaa, whereas in the three other structures, 8EYU, 8EYV, and 8EYW, in one monomer, the gcs of the first block are sssa, and in the second monomer ssaa.
* See the discussion about this topology in the main text.

A

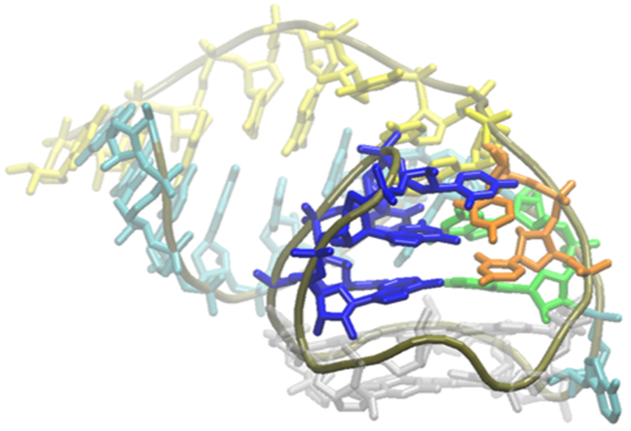

Parallel G4-RNA
(5BJO)

B

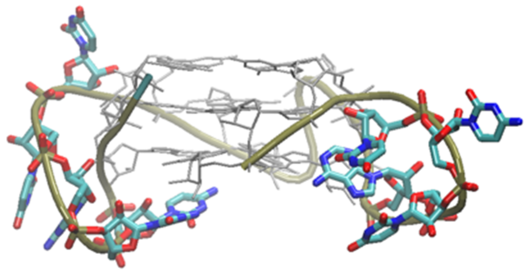

Parallel G4-RNA
(7Q48)

Figure S2. Structures of two parallel G4-RNAs. Under each structure, its topology and PDB ID are given. The stem is drawn as gray, transparent sticks (A) or thin sticks (B). The 5' extremity of the main chain tube is in cyan. In (A), the central 5-nt propeller loop is in dark blue, and the last 5'-FNs and the first 3'-FNs, which establish WC interactions with the central loop, are in green and orange, respectively. The rest of the 5'-FNs are in cyan and 3'-FNs in yellow. (B) The color code for the two long propeller loops is as follows: C: cyan, N: blue, O: red, and P: brown. Hydrogen atoms are omitted. As observed, the loops are long and unstructured.

Table S3. Number of parallel structures when at least one loop consists of 1 nt. Only the one-block structures are considered, from which a certain number of structures are excluded: the 20 htel G4s resolved in a crowding agent like PEG, because their topology is due to this agent, not to intrinsic reasons, the 12 hybrid4 with a 0-nt loop, the antiparallel-chair structure (8PSI) with a 5'-bottom snapback, the parallel structure with a D-loop (2M53) because of their particular constructions, the only LH one-block structure (6FQ2) and the structure with a 1-nt lateral loop (5J05). "1" means the loop length equals 1 nt, "−" means any loop length, including 1 nt, "≠1" means any loop length, excluding 1 nt.

Loop 1	Loop 2	Loop 3	Number of parallel G4s / Total number of G4s with the property
1	−	−	95 / 98 (97%)
−	1	−	58 / 64 (91%)
−	−	1	79 / 101 (78%)
1	≠1	≠1	3 / 5 (60%)
≠1	1	≠1	12 / 15 (80%)
≠1	≠1	1	1 / 20 (5%)
1	1	≠1	16 / 17 (94%)
1	≠1	1	48 / 48 (100%)
≠1	1	1	2 / 5 (40%)
1	1	1	28 / 28 (100%)

A **Theoretically**, if the loop is completely stretched and straight:
Minimum total length needed (3.42 x 2) + 21 = **28 Å**
where 3.42 Å is the average rise of two successive guanines in the stem

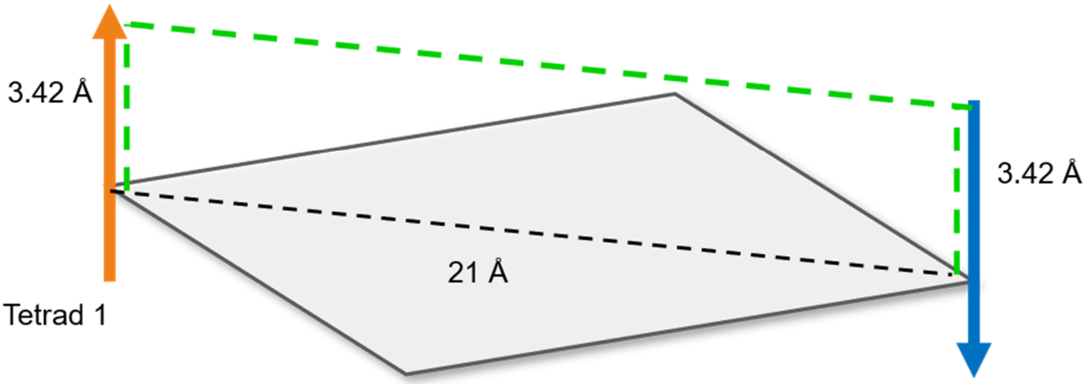

B **Experimentally**, on average, the distance between two consecutive nucleotides is 6.25 Å
(6.25 X 4) = **25 Å**
3 Å too short

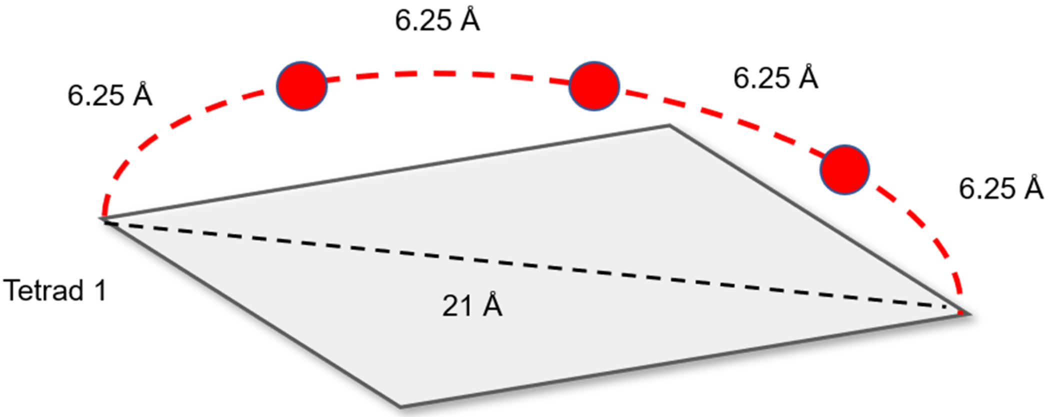

Figure S3. Demonstration for the need for more than 3 nts to form a diagonal loop. (A) Theoretically, the minimum length needed (28 Å), if the loop were straight and stretched (represented schematically by the green dashed line), although such a loop does not exist in nature, where it should be longer to keep its flexibility. (B) The loop length (25 Å) considering the average distance between two successive C5' atoms as observed in our set of 353 structures. In the NMR structure 143D, the total length is ~26 Å, which is still 2 Å too short, and leads to a non-planar first tetrad, with a distance between C5' atoms of opposite guanosines equal to 19 Å.

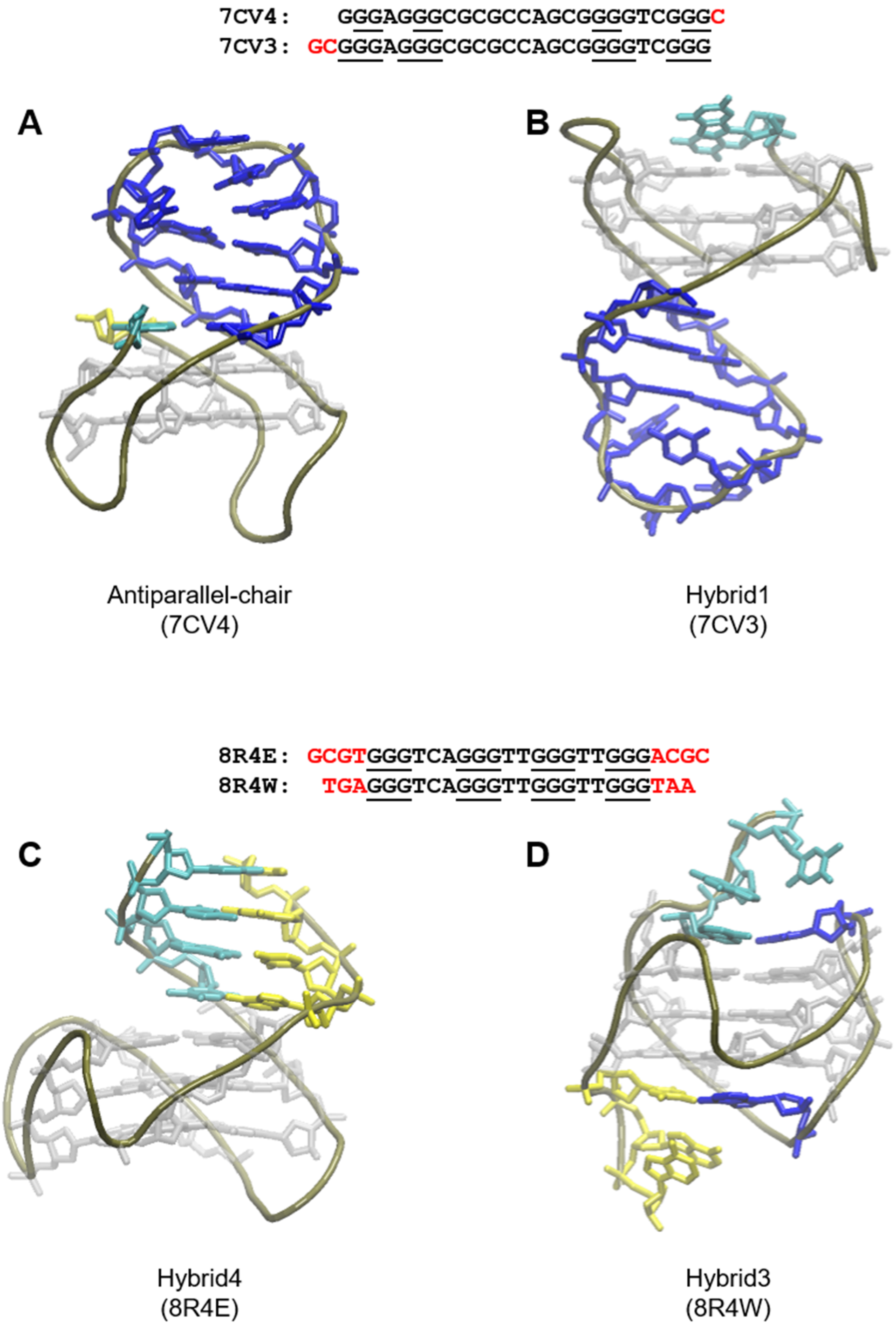

Figure S4. Small modifications in short FNs can produce important conformational changes. (A) → (B) and (C) → (D). The modified nts in the sequences are colored red, and Gs in the tetrads are underlined. The structures' color code: the stem is in transparent gray, the 5'-FN in cyan, the 3'-FN in yellow, and the loop nts forming WC interactions in dark blue.

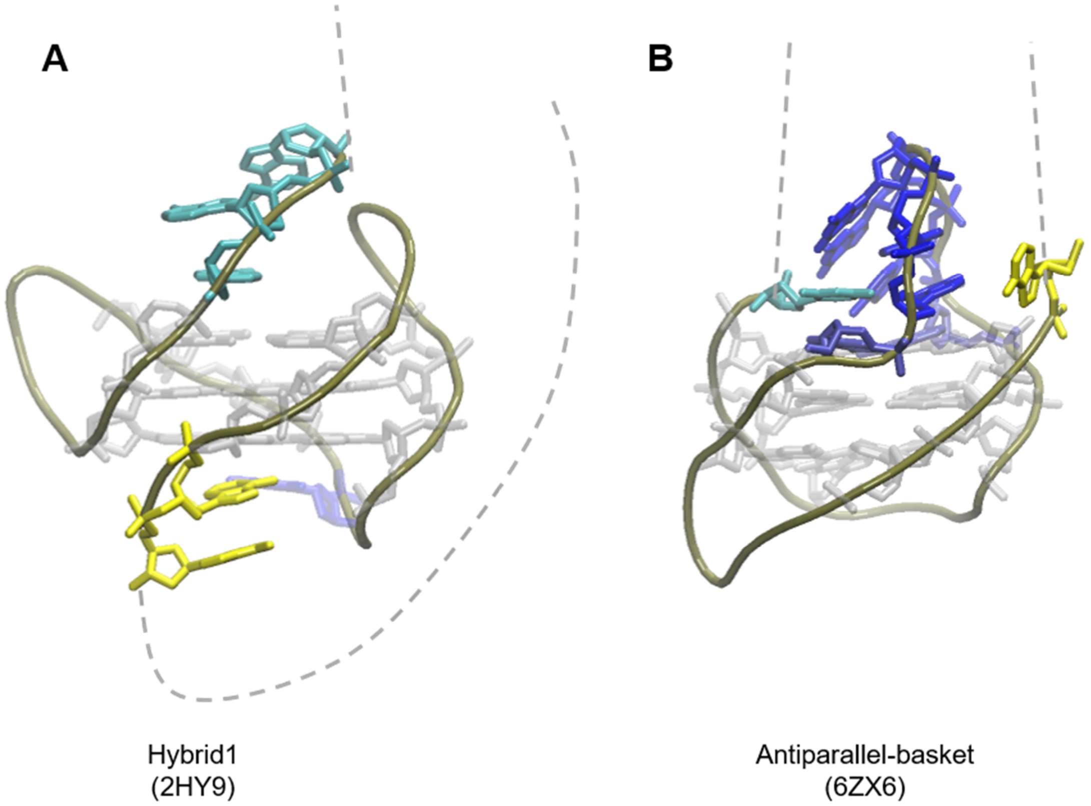

Figure S5. Exploration of the possible presence of long FNs. The putative WC-bp-forming FNs are drawn as gray dashed lines because they are considered difficult or improbable. Color code: The stem is in transparent gray, the 5'-FN in cyan, the 3'-FN in yellow. In (A), the thymine in the loop, which establishes WC interactions with the first adenine of the 3'-FN, is shown in transparent blue. In (B), the diagonal loop is in dark blue.

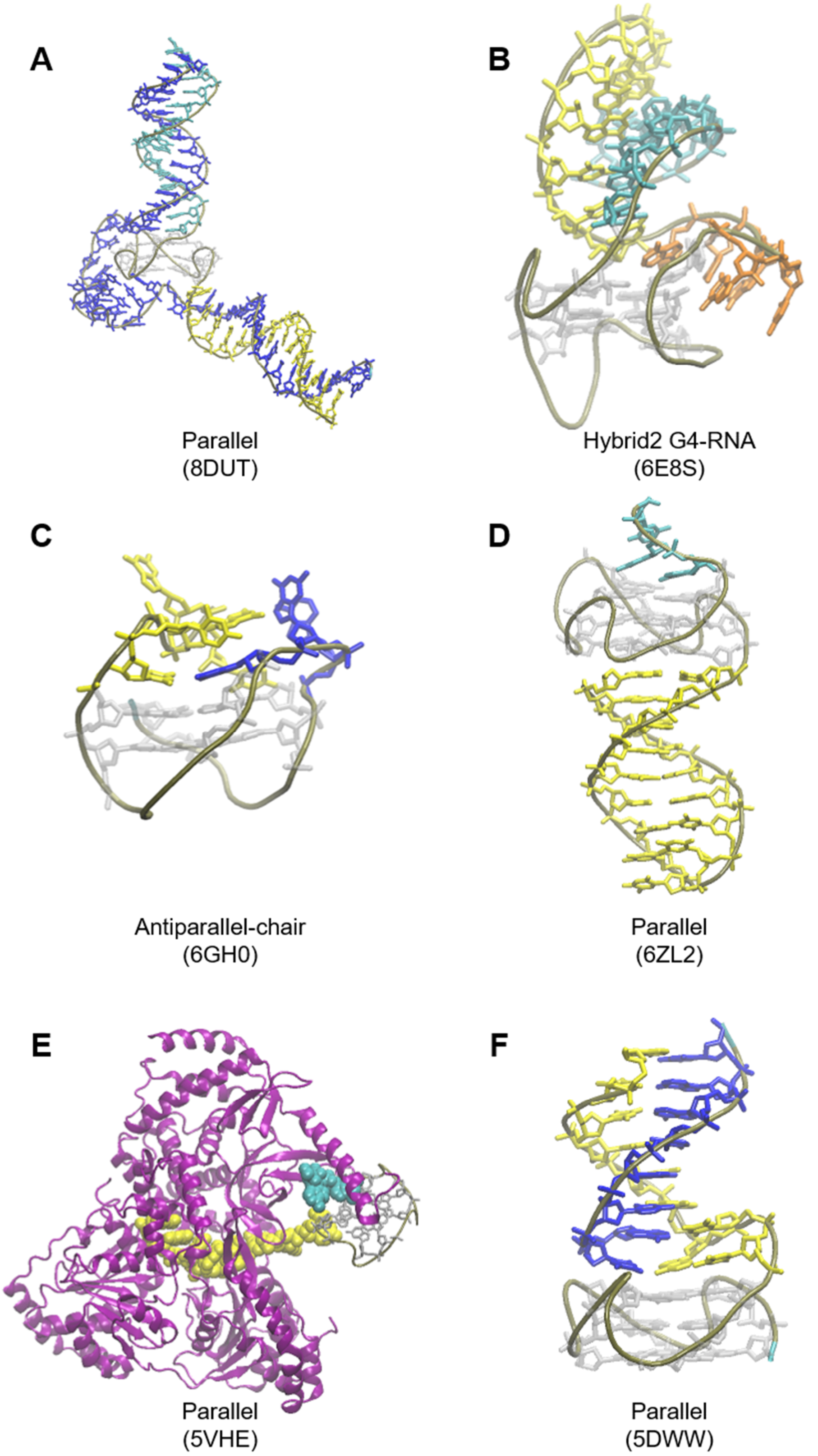

Figure S6. Various ways for stabilizing long FNs in the one-block G4s. (A-F) The stem is in transparent gray, the 5'-FN in cyan, the 3'-FN in yellow. In (A) and (F), the additional peptide is in dark blue, like the loop in (C). In (B), the small hairpin within the 3'-FN is in orange. In (E), because of the presence of helicase DHX36 (purple), the 5'-FNs and 3'-FNs are in hard spheres, and the stem is in solid gray sticks. In (F), the structure forms a stacking-stem dimer, but only one monomer with its additional nucleotide chain is shown.

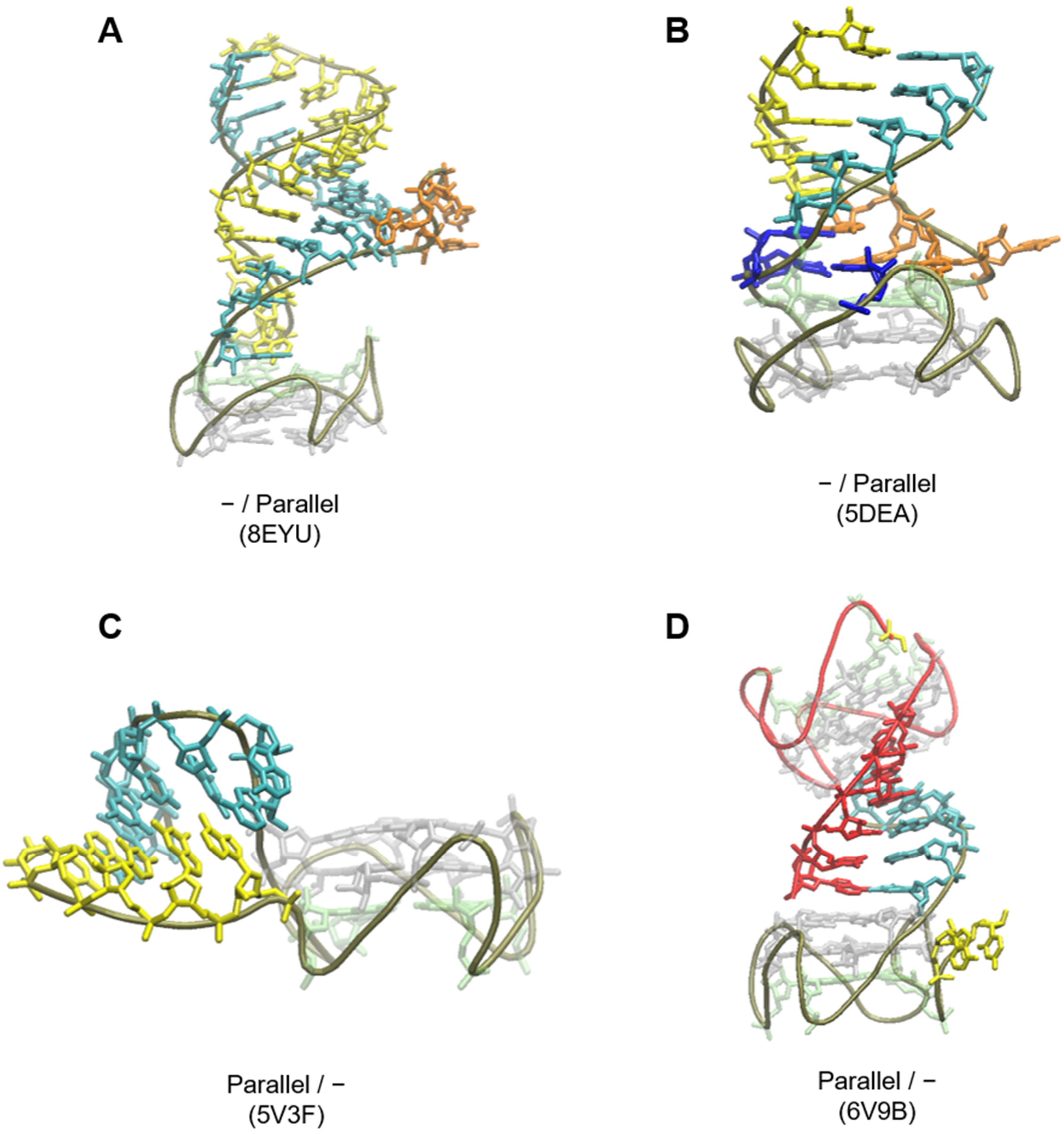

Figure S7. Various ways for stabilizing long FNs in the 3-tetrad two-block G4-RNAs. The topology is either −/parallel (A) and (B) or parallel/− (C) and (D), where "−" means the absence of topology because of the presence of only one tetrad (in transparent green), while the other block is parallel, made of two tetrads (in transparent gray). In (A) and (B), the structures form dimers, either a stacking-stem dimer (A) or not (B), but only one monomer is shown. In (D), exceptionally, the dimer is presented because it is needed for the stabilization of the 5'-FN. The second monomer has a red backbone and red 5'-FN. (A-D) The 5'-FN is cyan, the 3'-FN yellow, and loops dark blue. In (A) and (B), the nts of the 3'-FN, which make an additional loop (A) or establish WC interactions with the facing loops (B), are in orange.
